# Identification of a novel histone phosphorylation prognostic signature in hepatocellular carcinoma based on bulk and single-cell RNA sequencing

**DOI:** 10.3389/fendo.2022.965445

**Published:** 2022-08-31

**Authors:** Lei Fan, Ling Xu, Shan Tian, Xin Zheng

**Affiliations:** ^1^ Department of Infectious Diseases, Union Hospital, Tongji Medical College, Huazhong University of Science and Technology, Wuhan, China; ^2^ Joint International Laboratory of Infection and Immunity, Huazhong University of Science and Technology, Wuhan, China

**Keywords:** hepatocellular carcinoma, epigenetic modifying, gene signature, overall survival, histone phosphorylation

## Abstract

**Background:**

Hepatocellular carcinoma (HCC) is the third leading cause of death in the world, characterized by high morbidity, poor prognosis and high mortality. Histone modifications regulate intracellular gene expression at the post-transcriptional level, and disturbances in the regulatory pattern of histone modifications at individual locus or across the genome can lead to tumorigenesis of HCC. In this study, we constructed a prognosis-related histone phosphorylation regulated (HPR) genes signature and elucidated whether HPR genes can predict overall survival in HCC patients.

**Methods:**

Differentially expressed genes were screened using TCGA, ICGC and GEO databases, and a new risk signature was constructed by univariate Cox regression and Lasso regression analysis. Predictive nomograms were established by multivariate Cox regression of risk scores and clinical parameters, calibration curve and decision curve analysis were used to evaluate the models. The ssGSEA methods were used to determine the effect of risk scores on the tumor immune microenvironment. Data for HCC single-cell RNA sequencing (scRNA-seq) have been downloaded from Gene Expression Omnibus (GEO) to understand the role of HPR genes in tumorigenesis.

**Results:**

Our analyses of nine HPR genes provided prognostic insights. Overall survival in the low-risk and high-risk groups was statistically higher, respectively (P<0.001). Cox regression analysis revealed that the risk score is a significant predictor of HCC outcomes (HR=2. 2.62, 95%CI: 1.248-5.514, P=0.011). In addition, a nomogram combining risk scores with TNM stages was constructed and tested from calibration curves and decision curves (AUC=0.780). MHC-class-I genes, iDCs, Macrophages, Tfh, Treg, Th2 were overexpressed in the high-risk group.

**Conclusion:**

HPR genes risk score is closely related to the prognosis of HCC, tumor immune process and tumor cell progression.

## Introduction

Hepatocellular carcinoma (HCC) is one of the most common aggressive malignant tumors. It ranks second in incidence and third in mortality among malignant tumors in the world, and is also the third leading cause of cancer-related death worldwide (1). HCC is a major concern facing the global today in health care, with nearly 800,000 new cases each year, its incidence is on the rise in worldwide (2–4). In a 2018 World Health Organization report, liver cancer was the second leading cause of cancer-related death after lung cancer (5, 6). Despite recent breakthroughs in the treatment of viral hepatitis, the incidence of HCC is expected to continue to rise. With the rapid development of genomics, although in the same clinical stage, there are still great differences in the molecular characterization of tumor cells. The clinical efficacy and prognosis of treatment also vary widely according to the molecular typing of different types of patients. Various of the prognostic biomarkers commonly used in clinical practice still not satisfactory due to the varying levels of heterogeneity in HCC. HCC is mainly treated by surgery, combined with chemotherapy, radiotherapy and intervention therapy (7, 8). Despite these multiple therapies, the prognosis of HCC patients is currently not favorable, and it is clinically necessary to actively construct a prognostic assessment model (9). The development of bioinformatics has greatly facilitated the screening of prognostic biomarkers in tumor patients. For example, Song et al. construct an endoplasmic reticulum stress-related genes (ERGs) signature to predict the overall survival (OS) of patients with HCC (10). A prognostic model of lysine acetylation modifications (LARs) was developed to aid in individual prognostic monitoring and clinical decision-making in HCC (Sun et al.) (11). Fan et al. studied the expression profile and prognostic value of histone deacetylation in glioma for precise prediction of glioma prognosis (12). However, studies on the prognostic value of HPR in HCC are limited.

In recent years, histone modifications related to tumor formation and development have gradually attracted the attention of epigenetic researchers, and the increasing number of results indicate that tumors are often caused by histone modifications that affect their occurrence and prognosis (13). The epigenetic modifications offer ideal mechanisms for regulating gene expression and metabolic reprogramming. Histone phosphorylation is one of many ways of post-translational modification of histones. Histone phosphorylation is mainly involved in DNA damage repair, chromatin condensation, gene transcription and other cellular processes, and plays an important role in cell proliferation, differentiation, apoptosis and other processes. Aberrant histone phosphorylation has been detected in breast, prostate and colorectal cancer (14). In patients with chronic viral hepatitis, phosphatase overexpression is considered a key early risk factor for HCC. Aurora kinases are accountable for the phosphorylation of most serine/threonine residues in histone H3 (15). Aurora A is commonly overexpressed in HCC patients and is related with higher tumor grade. Activating NF-κB signaling through Aurora A reduces radiotherapy-induced apoptosis, thus preventing HCC from succumbing to radiation. A direct phosphorylation of histone H3 at Ser10 and Ser28 occurs after Aurora B interactions with histone H3, leading to chromosome instability and mitotic chromosome condensation (16, 17). Aurora B is considered to be an independent molecular marker for predicting tumor growth and invasion in HCC (18). Several preclinical studies have shown that inhibition of Aurora B can inhibit tumor growth and induce apoptosis in different cancers (19, 20). Current studies have shown that histone phosphorylation promotes the development of alcoholic liver disease. Ethanol and its metabolites increase histone H3 phosphorylation at serine 10 (H3S10) and serine 28 (H3S28) in primary rat hepatocytes by activating p38 mitogen-activated protein kinase (MAPK).

In this study, leveraging multi-omics data, we focused on identifying prognosis-related HPR genes and constructing an HPR-based genes signature, which was then validated for robustness with two external patient cohorts. In addition, the immune status of high-risk and low-risk groups was compared. We anticipate that this novel genetic signature may contribute to an independent prognostic indicator for HCC patients and facilitate the development of effective individualized therapy in the clinical setting.

## Material and methods

### Data screening and processing of bulk RNA-Seq data

HTSeq-FPKM data were obtained from The Cancer Genome Atlas data portal (https://portal.gdc.cancer) for HCC samples, along with details about clinical information. The International Cancer Genome Consortium (ICGC) data portal (https://dcc.icgc.org/) also provided clinical and mRNA expression data for the Japanese cohort of HCC (n = 231). At the same time, the dataset from the GEO website (https://www.ncbi.nlm.nih.gov/geo/) GSE14520 contains 221 United States cohort of HCC mRNA expression data and clinical information were also downloaded and used.

HPR-related genes were obtained from Gene Ontology Database (http://geneontology.org/) and Molecular Signature Database (MSigDB) (https://www.gsea-msigdb.org/gsea/msigdb/).After integration and deduplication, we established an optimized 47-gene HPR genes set. With the “limma” package in R, we identified HPR differentially expressed genes (DEGs) between HCC and normal tissue, based on FDR <0.05 and fold change >2. In parallel, univariate Cox regression was conducted to measure the correlation between each DEG and survival rate, and the genes showing a p value < 0.05 was considered a prognostic factor. Finally, differentially expressed HPGs associated with prognosis were obtained.

### Acquisition and processing of single-Cell RNA-Seq data

The single-cell RNA-seq dataset GSE149614 for HCC was obtained from the GEO website, including 10 patients with primary tumors and 8 patients with normal liver tissue, and subsequent analyses were performed. Use the R package Seurat (21) (version:4.0.2) workflow to process single-cell data, normalize the data, find 2000 highly variable genes, perform PCA dimensionality reduction through highly variable genes, select the top 20 PCs and use the harmony package to multi-group data integration, and unified manifold approximation and projection (UMAP) is applied to perform dimensionality reduction and visual clustering of cells. By calculating mitochondrial gene content and the number of genes expressed in each cell, cells with mitochondrial gene expression less than 20% and the number of genes detected Between 500 and 7000 cells were retained, leaving 62737 cells after filtration. Finally, we used the FindAllMarkers function to screen for the fold change greater than 2(FC>2) and the expression ratio of the genes (Minpct = 0.5) to calculate the marker genes of the 25 subgroups, and then used the corrected p < 0.05 to screen the marker genes. The marker genes were matched with the cell groupings through the cell marker website, and the singleR package was used to assist in judging the cell groupings. Finally, 25 cell groups were identified.

We then extracted normal hepatocytes as well as cancer cells to construct normal cell-to-tumor cell trajectories using Monocle 2 (version: 2.10.1) (22). Results were visualized using the Plot Cell Trajectories and Plot Complex Cell Trajectories functions using highly variable genes for trajectory inference, and annotated with cell types and calculated cell states. Once the cellular change trajectories are defined, we examine the expression changes of candidate genes in the trajectories.

### Construction and validation of the prognostic signature

First, the differential genes were subjected to univariate cox regression to obtain HCC-related prognostic genes. In order to prevent overfitting, Lasso regression analysis was performed through the “glmnet” package in the R software to construct a prognostic signature. According to the corresponding genes in the Lasso Cox regression model, the coefficients and their expression levels yield the risk score for each patient: Score = e sum (expression of each gene × corresponding coefficient). A risk score is available for each patient, and based on the median, patients can be divided into two groups. Kaplan-Meier curve analysis assessed survival differences between the two groups. To visualize these two groups, we performed Principal Component Analysis (PCA) and t-distributed Stochastic Neighbor Embedding (t-SNE) using the “Rtsne” R package. Finally, the prognostic predictive value of the model was validated with the ICGC cohort and GEO cohort.

### Visualization and assessment of prognostic value

We performed univariate and multivariate Cox regression analyses for multiple indicators that may affect survival outcomes, including risk score, sex, age, histological grade, AFP, fibrosis, and tumor stage. Based on multiple regression analysis, multiple predictors were integrated to construct a predictive nomogram model for OS. Calibration plots were used to compare the similarity between the 1-, 3-, and 5-year OS predicted using the nomogram with the idealized curves. The clinical utility of the predictive model was assessed using decision curve analysis.

### Evaluation of immune status

To examine the link between risk scores and patient immune status, single-sample genomic enrichment analysis (ssGSEA) was performed to compare 16 distinct immune cell subsets and 13 immune function pathways in two risk cohorts using “GSVA” package in the R software. Boxplots created using the R packages “pRRophetic” and “ggplot2” visualize the results of the high-risk and low-risk cohorts.

### Statistical analysis

Statistical analysis and data visualization were performed using R (version 4.1). Using the Wilcoxon test, differential genes were compared between HCC and matched non-tumor tissue. Kaplan-Meier analysis was used to estimate differences in survival between patients in different risk groups. Univariate and multivariate Cox regression analyses were used to screen for independent predictors of OS. For the “time-ROC” R package, the receiver operating characteristic (ROC) is used to evaluate the predicted probability of the risk model. In different risk groups, ssGSEA enrichment scores were compared between high and low risk groups by Mann-Whitney test. Pearson’s correlation was used to calculate whether there was a significant correlation between risk scores and active immunization pathways. Statistical significance was determined by P-value and false discovery rate less than 0.05.

## Result

### Patients’ characteristics

**Figure 1:**
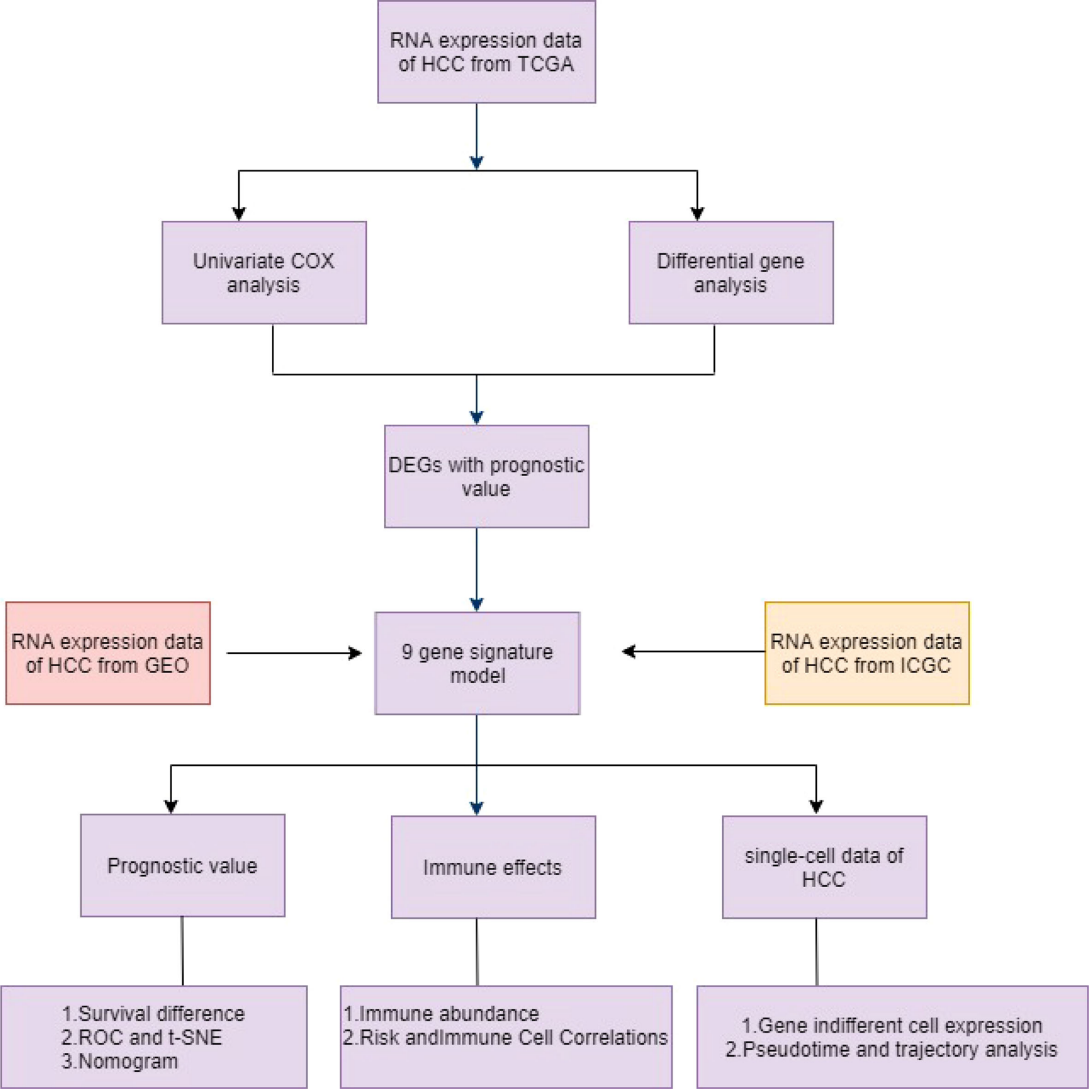
Schematic diagram of the overall design of the study.

In this study, the TCGA database contains 377 HCC patients, only 374 patients have mRNA expression data, and 370 of these 374 patients have complete survival time and survival status data. 231 HCC samples in the ICGC dataset and 221 HCC samples in the GEO dataset were included as the validation cohort and data collected in December 2021. The completed statistical characteristics of the cases are shown in **Table 1**, and the completed flow chart of this study is shown in **Figure 1**.

**Table 1:** Clinicopathological parameters of the HCC patients.

Characteristic	TCGA cohort	ICGC cohort	GEO cohort
number of patients	370	231	221
Age at diagnosis (years)	59 (16-90)	67 (31-89)	51 (21-77)
Gender			
Male	249 (67.3%)	170 (72.6%)	30 (13.6%)
Female	121 (32.7%)	61 (26.4%)	191 (86.4%)
Stage	BCLC	LCSGJ	BCLC
I	171 (46.2%)	36 (15.6%)	93 (42.1%)
II	85 (23.0%)	105 (45.5%)	77 (34.8%)
III	85 (23.0%)	71 (30.7%)	49 (22.2%)
IV	5 (1.3%)	19 (8.2%)	0
Unknown	24 (6.5%)	0 (0.0%)	2 (0.9%)
T classfication			
T1	181 (48.9%)	NA	NA
T2	93 (25.2%)	NA	NA
T3	80 (21.6%)	NA	NA
T4	13 (3.5%)	NA	NA
Unknown	3 (0.8%)	NA	NA
N classfication			
N0	252 (68.1%)	NA	NA
N1	4 (1.1%)	NA	NA
Unknown	114 (30.8%)	NA	NA
M classfication			
M0	266 (71.9%)	NA	NA
M1	4 (1.1%)	NA	NA
Unknown	100 (27.0%)	NA	NA
Tumor grade			
Grade 1	55 (14.9%)	NA	NA
Grade 2	177 (47.8%)	NA	NA
Grade 3	121 (32.7%)	NA	NA
Grade 4	12 (3.2%)	NA	NA
Unknown	5 (1.4%)	NA	NA
HBV viral status			
AVR-CC	NA	NA	56 (25.3%)
CC	NA	NA	156 (70.1%)
N	NA	NA	6 (2.7%)
Unknown			
Main Tumor Size (>/<=5 cm)			
>5 cm	NA	NA	80 (36.2%)
<=5 cm	NA	NA	140 (63.3%)
Unknown	NA	NA	1 (0.4%)
AFP (>/<=300ng/ml)			
>300ng/ml	66 (17.8%)	NA	100 (45.2%)
<=300ng/ml	218 (58.9%)	NA	118 (53.4%)
Unknown	86 (23.2%)		3 (1.4%)
Survival status			
Alive	240(64.9%)	189 (81.8%)	136 (61.5%)
Deceased	130(35.1%)	42 (18.2%)	85 (38.5%)

NA, not available; AVR-CC, active viral replication chronic carrier; CC, chronic carrier; N, no HBV; AFP, alpha fetoprotein.

### Differentially expressed genes related to histone phosphorylation

**Figure 2:**
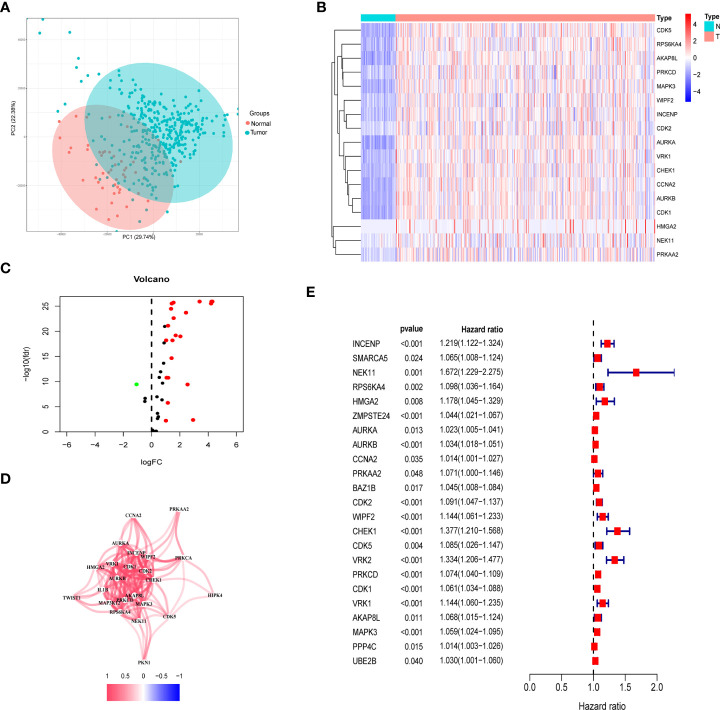
Identification of prognostic histone phosphorylation-related genes in the TCGA cohort. **(A)** PCA of normal and tumor samples. **(B)** The heatmap of DE-HPGs in the TCGA cohort; N represent normal and T represent tumor. **(C)** The volcano-plot of DE-HPGs; red denotes up-modulated genes and blue denotes down-modulated genes. **(D)** The correlation network of candidate genes. **(E)** Univariate Cox regression analysis identified prognostic variables for HR with 95% CI and P-value. PCA, principal component analysis; DE-HPGs, differentially expressed histone phosphorylation-related genes.

Among the TCGA dataset, principal component analysis shows that tumor samples and normal samples are clustered together (**Figure 2A**). First, we extracted the histone phosphorylation expression matrix, according to the screening criteria, a total of 23 genes showed differential expression in HCC compared with paracancerous tissues, including 22 up-regulated and 1 down-regulated. The heat map (**Figure 2B**) and volcano map (**Figure 2C**) show the distribution of HPGs. Interactions between these prognostic genes are shown in correlation networks (**Figure 2D**). Combining differential genes with patient survival information, univariate COX risk regression showed that 17 genes were associated with survival outcomes of HCC individuals (**Figure 2E**).

### Development and validation of histone phosphorylation gene signature

**Figure 3:**
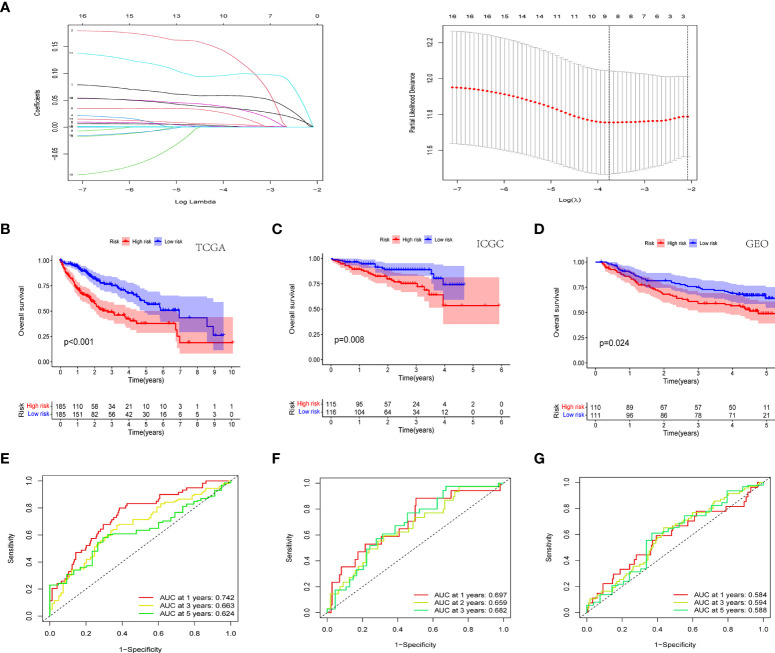
Novel histone phosphorylation-related genes risk signature. **(A)** LASSO regression screening model genes. Survival analysis of high- and low-risk groups in the TCGA cohort **(B)** ICGC cohort **(C)** and GEO cohort **(D)**. ROC curve analysis for predicting overall survival in HCC patients based on risk scores in the TCGA cohort **(E)** ICGC cohort **(F)** and GEO cohort **(G)**.

In order to ensure that an effective prognostic model can be predicted, the 17 prognostic genes of univariate analysis were compressed in the training group using LASSO regression to prevent overfitting, and 9 genes were obtained to build a model. (**Figure 3A**) (**Table2**). Calculate the risk score of each patient according to the corresponding gene coefficient and its expression level: Risk Score = (0.06 * INCENP expression level) + (0.13 * NEK11 expression level) + (0.02 * AURKB expression level) + (0.001 * CCNA2 expression) + (0.022 * PRKAA2 expression level) + (0.098 * CHEK1 expression level) + (0.035 * CDK5 expression level) + (0.035 * PRKCD expression level) + (0.05* CDK1 expression level). Next, Kaplan-Meier curves were used to evaluate the survival rates of different groups of patients, and the low-risk group showed higher survival rates (p<0.001), a prognostic value exists for the risk score (**Figures 3B-D**). ROC curves were widely used to assess the sensitivity and specificity of the model, and the accuracy of the histone phosphorylation gene signature in predicting 1-, 3-, and 5-year OS in our model was 0.742, 0.663, and 0.624 (**Figures 3E-G**), respectively, indicates a good predictive power.

**Table 2:** Detail information of the prognostic gene signatures.

Gene	Coefficient	Gene name
INCENP	0.06	Inner centromere protein
NEK11	0.13	Serine/threonine-protein kinase Nek11
AURKB	0.02	Aurora kinase B
CCNA2	0.001	Cyclin-A2
PRKAA2	0.022	Protein kinase AMP-activated catalytic subunit alpha 2
CHEK1	0.098	Serine/threonine-protein kinase Chk1
CDK5	0.035	Cyclin-dependent kinase 5
PRKCD	0.035	Protein kinase C delta type
CDK1	0.05	Cyclin-dependent kinase 1

**Figure 4:**
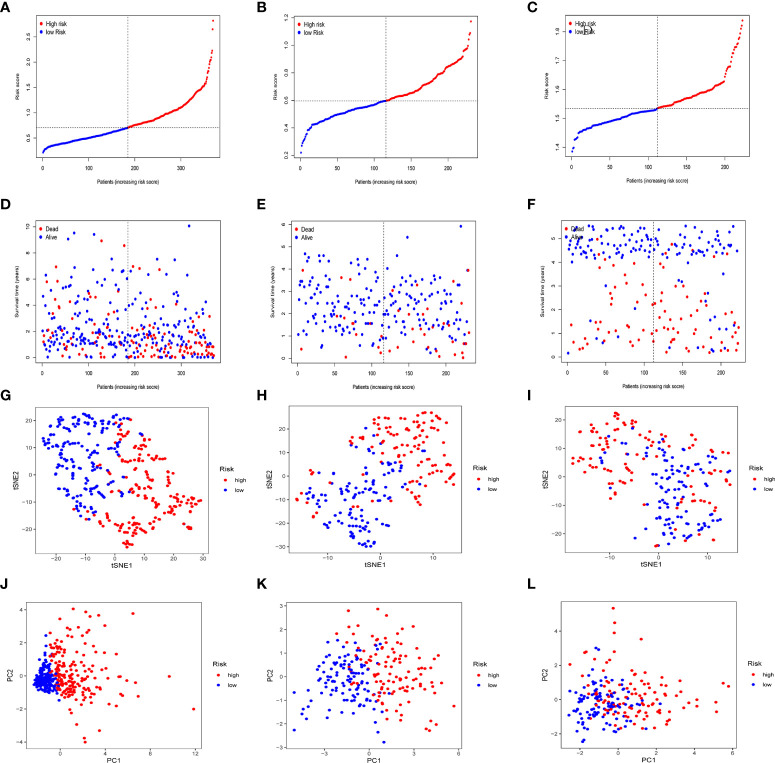
Prognostic analysis of prognostic models in the TCGA cohort, the ICGC cohort and GEO cohort. Patients’ survival status and risk score in the TCGA cohort **(A, D)**, ICGC cohort **(B, E)** and GEO cohort **(C, F)**. PCA analysis and t-SNE analysis of high and low risk groups in the TCGA cohort **(G, J)**, ICGC cohort **(H, K)** and GEO cohort **(I, L)**.

In the TCGA cohort, ICGC cohort, and GEO cohort, all patients were divided into high and low risk groups using median risk values. The distribution of risk scores and patient survival status in different groups showed that HCC patients in the high-risk group had a shorter survival time and a higher incidence of death (**Figures 4A-F**). Dimensional reduction of patients by risk score was visualized, PCA and t-SNE analysis showed that the same risk group was clustered together (**Figures 4G-L**).

### Construction of the nomogram

**Figure 5:**
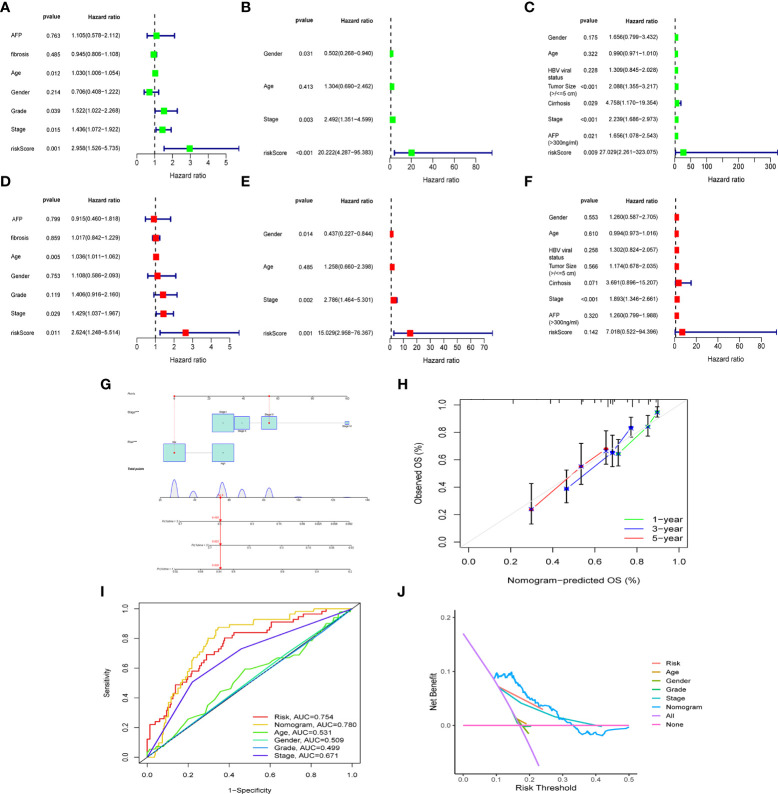
The construction of OS predictive nomogram for HCC patients. Results of univariate and multivariate Cox regression analysis of OS TCGA cohorts **(A, D)**, ICGC cohorts **(B, E)** and GEO cohorts **(C, F)**. Univariate Cox regression analysis to screen for OS-related factors **(A-C)**. Multivariate Cox regression analysis to screen for OS-related factors **(D-F)**. The nomogram is used for predicting 1-, 3-, and 5-year overall survival probability of HCC patients **(G)**. The calibration plots for predicting patient 1-, 3-, or 5-year OS **(H)**. AUC of clinical characteristics, risk score, and the risk score combined with tumor stage at 1-year OS **(I)**. DCA curves for two independent prognostic factors or a combination of them in OS prediction **(J)**. HCC, hepatocellular carcinoma; OS, overall survival; ROC, receiver operating characteristic curve; DCA, decision curve analysis; AUC, area under curve.

To further confirm whether risk score can serve as an independent prognostic factor for HCC, we performed multivariate and univariate Cox regression analysis together with possible clinical indicators, including TNM stage, gender, age, histological grade, AFP, fibrosis and risk score. As shown in **Figure 5A-F**, based on multivariate Cox regression analysis, risk score and clinical stage emerged as independent prognostic factors in the TGCA cohort (TCGA: HR = 2.62, 95% CI = 1.25–5.51, p =0.011). In order to obtain individualized assessment for each patient, we constructed nomograms of the variables obtained from the multivariate prognostic analysis, and display the constructed nomogram on the calibration curve and the ideal model can achieve similar results (**Figures 5G-H**). By combining the two independent prognostic factors, risk score and clinical stage, a significant improvement in mortality at 1-, 3-, or 5-year OS was seen (AUC=0.78) (**Figure 5I**). Decision Curve Analysis (DCA) has also shown that predictive models have good clinical utility (**Figure 5J**).

### Correlation between risk score and clinical characteristics

**Figure 6:**
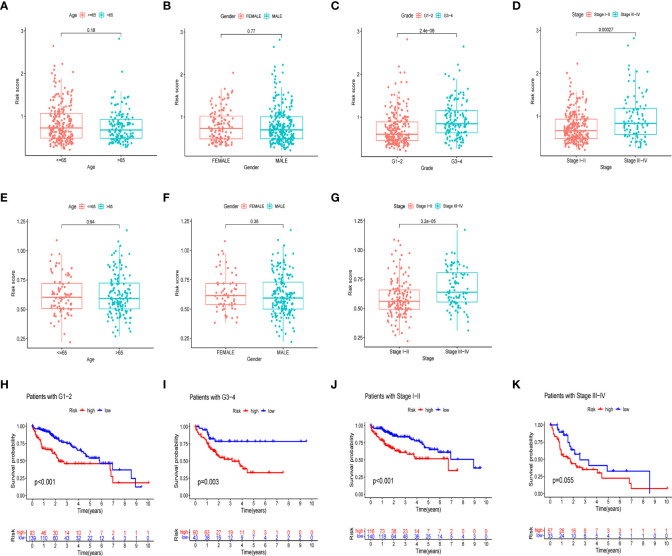
Risk scores and survival curves of different groups stratified by clinical characteristics. TCGA cohort **(A-D)**, ICGC cohort **(E-G)**. **(A, E)** Age, **(B, F)** gender, **(C)** tumor grade, **(D, G)** tumor stage. **(H)** survival curves of Grade1-2. **(I)** survival curves of G3-4. **(J)** survival curves of stage I-II **(K)** survival curves of stage III-IV.

We further investigated the clinical utility of the HPR risk score, examining the relationship between the risk score and clinicopathological features (including tissue grade, TNM stage, gender, and age). **Figures 6A-G** depict the distribution of risk scores between groups by clinical characteristics. Based on these data, we found that risk scores were independent of age and sex (p>0.05) (**Figures 6A, B, E, F**), whereas higher risk scores were found in patients with advanced TNM stage or higher pathological grade, and the ICGC cohort came to the same conclusion. In addition, **Figures 6H-K** show differences in survival between high and low risk groups at the same stage and grade. Among patients of the same grade and stage, survival analysis showed that the high-risk groups all had lower survival times. It indicated that the progression of HCC was related to HPR.

### Immune status between two risk groups

**Figure 7:**
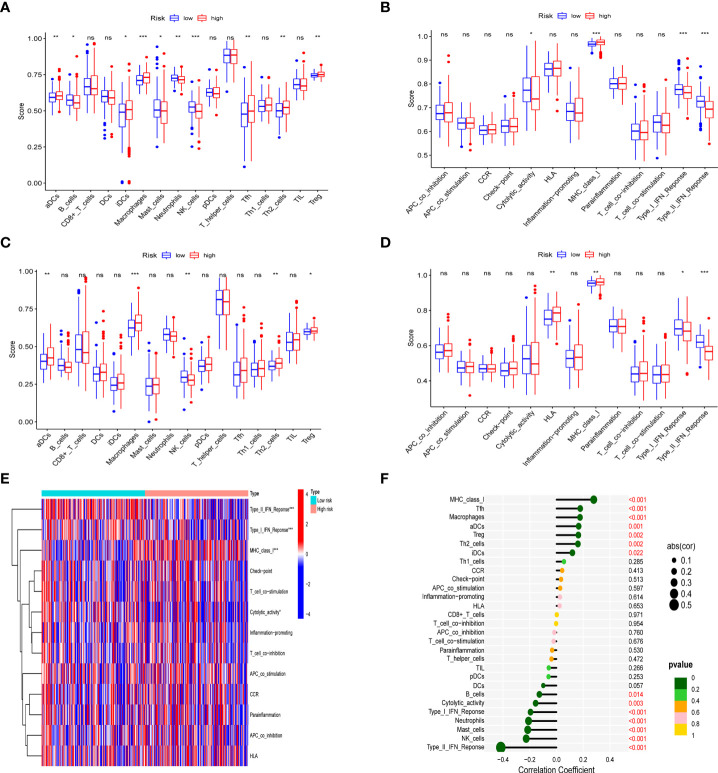
Analysis of immune status in different risk groups. Comparison of the ssGSEA score of immune infiltrating cells and immune-related functions in different risk groups. TCGA cohort **(A, C)**, ICGC cohort **(B, D)**. **(E)** The heatmap of immune-related functions in different risk groups in the TCGA cohort **(F)** The lollipop plot depicting the link between immune infiltrating cells and risk scores; node size represents correlation coefficient; node color represents P-value. *p < 0.05, **p < 0.01, ***p < 0.001; ns, no significant.

In different risk groups, we measured enrichment scores for 16 immune cells and 13 immune functions using the ssGSEA algorithm. According to the results, antigen-presenting cells such as aDC, Th2, Treg, and macrophages were enriched in the high-risk group (**Figures 7A, B**). In addition, immune function levels including MHC class I were also enriched in the high-risk group, while NK cells, B cells, Type-II-IFN response, and Type-I-IFN response were enriched in the low-risk group (**Figures 7C-E**). Furthermore, the presence of immune cells in the tumor microenvironment was positively associated with the risk score (including Tfh cells, aDC cells, Treg cells, Th2 cells and macrophages) and with immune function (MHC_class_I). However, NK cells, B cells, Type-II-IFN response, and Type-I-IFN was negatively correlated with the risk score shown in the lollipop plot (**Figure 7F**). This difference in immune infiltration may account for poor prognosis in high-risk patients.

### Validation of differential expression of prognostic genes in single-cell RNA-Seq data

**Figure 8:**
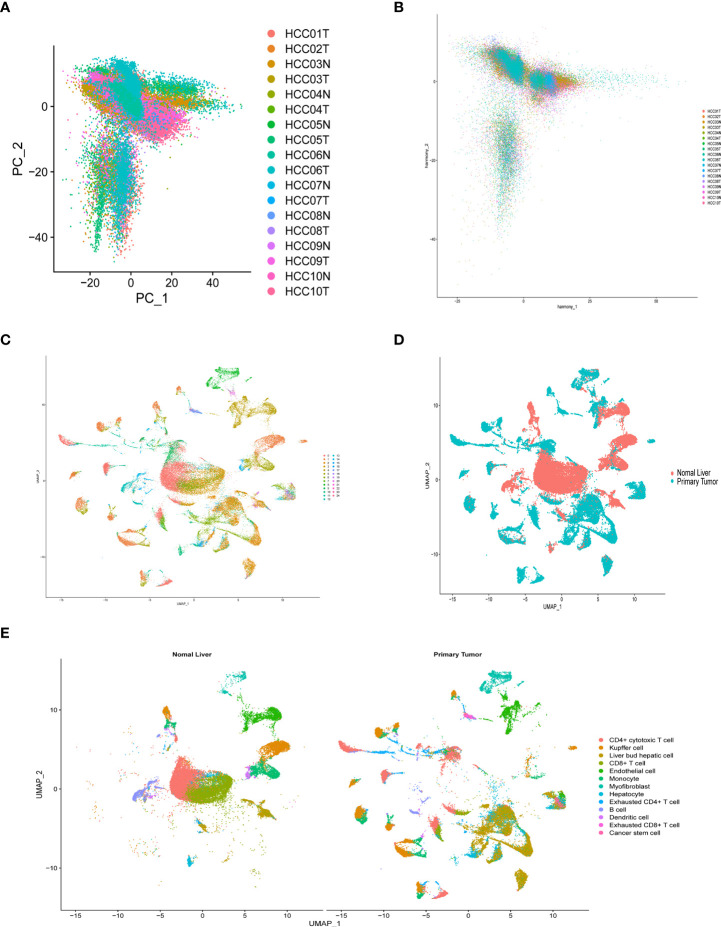
Overview of single cells from tumor samples and normal samples. **(A)** dimensionality reduction graph of two different types of samples. **(B)** Multi-data integration to remove batch effects of two different types of samples. **(C)** UMAP of the 25 cell clusters. **(D)** The sample types of the cells. **(E)** The cell types were identified by marker genes.

**Figure 9:**
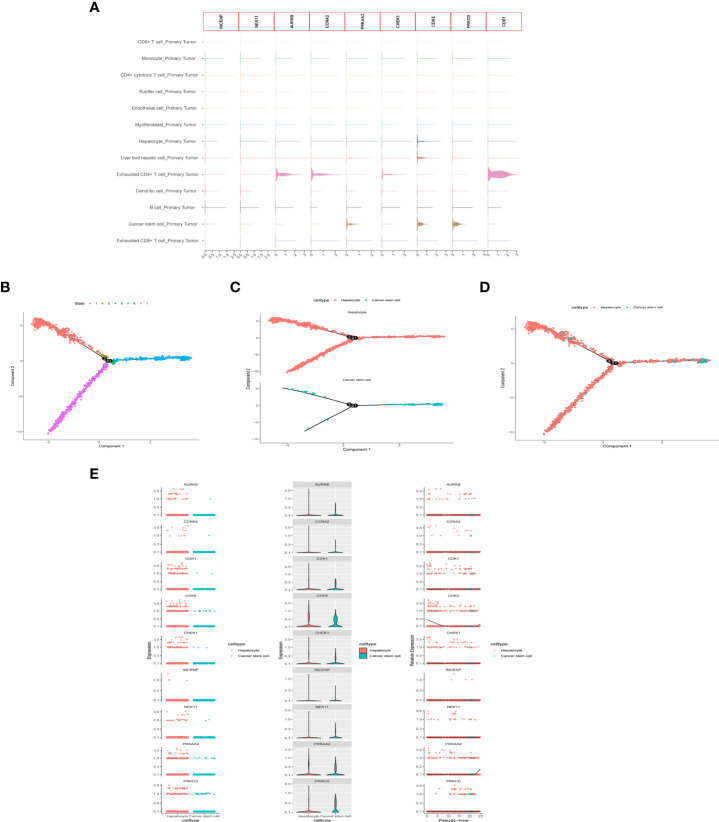
scRNA-seq Data processing and analysis. **(A)** Expression of the 9 genes in tumor tissue. **(B-D)** Pseudotime and cell trajectory analysis. **(E)** Changes of the 9 genes in cell trajectory analysis.

After quality control and normalization of the dataset GSE136103, data for 21,560 genes and 62737 cells were retained. Using the umap algorithm, 62737 cells were divided into 25 clusters. We obtained human cell marker genes from CellMarker (http://biocc.hrbmu.edu.cn/CellMarker/), and based on the marker genes and the R package “singleR” to help mark cell grouping, we annotated these 25 clusters (clusters 0 and 9 are CD4+ cytotoxic T cells; clusters 1, 23 are Kupffer cells; clusters 2, 6, 19, 21, 24 are Liver bud hepatic cells; clusters 3, 7 are CD8+ T cells; clusters 4, 20 are Endothelial cells, Cluster 5 is Monocyte cell, Cluster 8 is Myofibroblast cell, Cluster 10, 13, 16, 22 is Hepatocyte cell, Cluster 11 is Exhausted CD4+ T cell, Cluster 12, 14 is B cell, Cluster 15 is Dendritic cell, Cluster 17 is Exhausted CD8+ T cells, cluster 18 are Cancer stem cells (**Figures 8A-E**). We then examined the expression of 9 model genes in single cells and found that CDK1, AURKB, CCNA2, and CHEK1 are mainly expressed in CD4 cells, while PRKAA2, CDK5, and PRKCD are mainly expressed in tumor cells (**Figure 9A**).

To identify the developmental relationship between hepatocytes and malignant cells, we executed single-cell trajectory analysis of scRNA-seq data using Monocle. We therefore defined the hepatocyte cluster as the root and the tumor cell cluster as the end of the trajectory (**Figures 9B-D**). As cells transition from one state to another, some genes are silenced while others become newly active. We found that PRKCD, PRKAA2, CDK5, and CDK1 genes were increased in the progression from hepatocytes to tumor cells (**Figure 9E**).

## Discussion

HCC is a common malignancy, and the widely used TNM staging system is often ineffective in predicting prognosis. Biomarkers that predict patients’ survival rates are urgently needed for HCC (23, 24). Various types of histone phosphorylation are involved in gene transcription, DNA repair, apoptosis and chromosome condensation, and play a crucial role in metabolic regulation and tumorigenesis (25). Here, using multi-omics HCC data from multiple cohorts, we performed an extensive bioinformatics analysis to build a risk model consisting of 9 HPR genes that were significantly associated with prognosis in HCC patients. By exploring the relationship between HPR score and clinical parameters in HCC patients, it was demonstrated that HPR was associated with high stage and grade, and immune infiltration was also analyzed to further investigate the clinical significance of prognosis. Finally, the expression of HPR genes in single-cell sequencing was analyzed, and the results also confirmed that most of HPR genes were highly expressed in tumor cells. This HPR risk model not only provides more intuitive information for clinicians to personalize the treatment of HCC in the future, but also provides a molecular basis for HPR.

In this study, a prognostic risk model for 9 genes INCENP, NEK11, AURKB, CCNA2, PRKAA2, CHEK1, CDK5, PRKCD, and CDK1 was established by univariate Cox and LASSO Cox regression selection. CDK5 and CDK1 is an atypical member of the CDK family, a serine/threonine kinase plays an important role in multiorgan tumorigenesis, activation of CDK5 is associated with HCC tumorigenesis. Hepatocyte proliferation and tumorigenesis are enhanced by CDK5-mediated phosphorylation and stabilization of TPX2. Cdk5 induces FAK phosphorylation at Ser732, a component of mitosis and spindle formation in tumor cells (26–28). Aurora-B is a chromosomal passenger protein that forms a complex with INCENP and survivin to regulate stable bipolar spindle-kinetochore attachment during mitosis, chromosome segregation and cytokinesis. Demonstrating that AURKB has been shown to be closely associated with liver cancer, its upregulation plays a key role in promoting hyper polyploidization, and an increase in AURKB phosphorylation was detected on intermediates during cytokinesis, leading to hyperpolyploidization (29–31).

They are serine/threonine kinases that are collectively known as the NIMA-related kinase (NEK) family. Members of the family include NEK1-NEK11. NEK11 is integral to the cell cycle and microtubule formation (32). The expression of PRKAA2 helps activate autophagy and chemoresistance. PRKAA2 plays a key role in regulating autophagy and 5-FU resistance in gastric cancer (33). CHEK1 is responsible for the control of G1/S, S and G2/M checkpoints, and PRKCD inhibits autophagy. phagocytosis to promote the proliferation, invasion and metastasis of hepatoma cells (34, 35). The cyclin family gene CCNA2 has been shown to be tumor-promoting in multiple solid tumor types, and its expression is differential between many types of cancer and normal tissues. Cancer of multiple types may be affected by CCNA2 (36–38).

The immune microenvironment plays an important role in tumorigenesis and has been widely recognized. The results of ssGSEA analysis showed that the high-risk group had higher infiltration levels of Treg, iDCs, macrophages and Th2 cells, and higher expression levels of MHC-class-I genes. Fu et al. showed that increased macrophages and Treg cells in HCC patients are associated with poor prognosis, and this is consistent with our findings in the high-risk group (39). Shankaran et al. elucidated that IFNγ and lymphocytes in the immune system can cooperate to suppress tumor development, consistent with our results with elevated B cells, Type-II-IFN response in the low-risk group (40). The results demonstrate the relationship between HPR and immunity, emphaizing the critical role of immunotherapy in high-risk HCC patients.

scRNA-seq has become an important tool for studying tumor heterogeneity and tumor cell evolution in a variety of cancers (41). Here, we used HCC scRNA-seq data from the GEO database to explore trajectory analysis from normal liver cells to tumor cells. INCENP and NEK11 genes were expressed at a low level in the single-cell transcriptome due to the limitation of single-cell technology. CDK1, AURKB, CCNA2, and CHEK1 were highly expressed in immune cells, suggesting that histone phosphorylation is closely related to the tumor immune environment. Cell trajectories showed that the expression of PRKCD, PRKAA2, CDK5, and CDK1 genes increased from hepatocytes to tumor cells. These findings identify the nine genes that play important roles in immune cell infiltration and in the development of normal cells into tumor cells, providing a basis for subsequent molecular studies.

This study may have several unavoidable limitations. First, we constructed a differentially expressed HPR-based prognostic model to predict survival in HCC patients based on data from the TCGA database. Although two external datasets were used as validation, it still needs to be validated in real clinical samples. Second, differentially expressed HPR genes need to be validated in molecular experiments. The last major limitation is the lack of in-depth mechanism research. Bioinformatics provides an analytical basis for screening genes, but how these genes cause histone phosphorylation needs to be further improved by *in vitro*/*in vivo* experiments.

## Conclusion

HCC is a common and high-burden tumor. We elaborated the role of HPGs in the prognosis and immune microenvironment of HCC. HP risk score has been identified as an important prognostic factor for HCC, improving the predictive power of the TNM staging system. Collectively, these findings provide new insights into the prognostic assessment and treatment of HCC.

## Data availability statement

The datasets presented in this study can be found in online repositories. The names of the repository/repositories and accession number(s) can be found in the article/**Supplementary Material.**

## Author contributions

LF designed the study and collected and analyzed data. LF and LX wrote and contributed to the manuscript. ST and XZ revised the manuscript. All authors contributed to the article and approved the submitted version.

## Funding

This work was supported by the National Science and Technology Major Project for Infectious Diseases of China (2018ZX10302206, 2018ZX10723203, and 2017ZX10304402-002-005); the Applied Basic and Frontier Technology Research Project of Wuhan (2020020601012233) the Innovation Team Project of Health Commission of Hubei Province [WJ2019C003].

## Conflict of interest

The authors declare that the research was conducted in the absence of any commercial or financial relationships that could be construed as a potential conflict of interest.

## Publisher’s note

All claims expressed in this article are solely those of the authors and do not necessarily represent those of their affiliated organizations, or those of the publisher, the editors and the reviewers. Any product that may be evaluated in this article, or claim that may be made by its manufacturer, is not guaranteed or endorsed by the publisher.
