# Supplementary material for: Identification of a novel histone phosphorylation prognostic signature in hepatocellular carcinoma based on bulk and single-cell RNA sequencing
Source: Front Endocrinol (Lausanne). 2022 Aug 31;13:965445. doi: 10.3389/fendo.2022.965445 (PMC9470838; doi:10.3389/fendo.2022.965445)
Supplement: Supplementary file 1 [file DataSheet_1.docx]

##########提取HPG基因表达量

library(limma)

#设置工作目录

setwd("E:\\tcgaGexp")

rt=read.table("symbol.txt",sep="\t",header=T,check.names=F)

rt=as.matrix(rt)

rownames(rt)=rt[,1]

exp=rt[,2:ncol(rt)]

dimnames=list(rownames(exp),colnames(exp))

data=matrix(as.numeric(as.matrix(exp)),nrow=nrow(exp),dimnames=dimnames)

data=avereps(data)

data=data[rowMeans(data)>0,]

gene=read.table("gene.txt", header=F, check.names=F, sep="\t")

sameGene=intersect(as.vector(gene[,1]),rownames(data))

geneExp=data[sameGene,]

out=rbind(ID=colnames(geneExp),geneExp)

write.table(out,file="tcga.HPGexp.txt",sep="\t",quote=F,col.names=F)

##############提取TCGA和ICGC数据集共有表达量

library(limma)

library(sva)

setwd("E: \\intersect")

#读取TCGA代谢基因表达文件,并对数据进行处理

rt = read.table("tcga.FRGexp.txt",header=T,sep="\t",check.names=F)

rt=as.matrix(rt)

rownames(rt)=rt[,1]

exp=rt[,2:ncol(rt)]

dimnames=list(rownames(exp),colnames(exp))

metab=matrix(as.numeric(as.matrix(exp)),nrow=nrow(exp),dimnames=dimnames)

metab=avereps(metab)

#读取ICGC基因表达文件,并对数据进行处理

rt = read.table("ICGCsymbol.txt",header=T,sep="\t",check.names=F)

rt=as.matrix(rt)

rownames(rt)=rt[,1]

exp=rt[,2:ncol(rt)]

dimnames=list(rownames(exp),colnames(exp))

geo=matrix(as.numeric(as.matrix(exp)),nrow=nrow(exp),dimnames=dimnames)

geo=avereps(geo)

#对基因取交集,分别输出交集基因在代谢矩阵和ICGC矩阵的表达量

sameGene=intersect(row.names(metab),row.names(geo))

metabOut=metab[sameGene,]

geoOut=geo[sameGene,]

all=cbind(metabOut,geoOut)

metabOut=rbind(ID=colnames(metabOut),metabOut)

write.table(metabOut,file="tcgaMetabExp.share.txt",sep="\t",quote=F,col.names=F)

geoOut=rbind(ID=colnames(geoOut),geoOut)

write.table(geoOut,file="ICGCMetabExp.share.txt",sep="\t",quote=F,col.names=F)

###################分析正常和肿瘤组织HPR差异基因并绘制热图

#引用包

library(limma)

library(pheatmap)

expFile="tcgaExp.txt" #基因表达文件

setwd("E:\\ Diff")

#读取输入文件

rt=read.table(expFile, header=T, sep="\t", check.names=F)

rt=as.matrix(rt)

rownames(rt)=rt[,1]

exp=rt[,2:ncol(rt)]

dimnames=list(rownames(exp), colnames(exp))

data=matrix(as.numeric(as.matrix(exp)), nrow=nrow(exp), dimnames=dimnames)

data=avereps(data)

#区分正常和肿瘤样本

group=sapply(strsplit(colnames(data),"\\-"), "[", 4)

group=sapply(strsplit(group,""), "[", 1)

group=gsub("2", "1", group)

conNum=length(group[group==1]) #正常组样品数目

treatNum=length(group[group==0]) #肿瘤组样品数目

sampleType=c(rep(1,conNum), rep(2,treatNum))

#差异分析

sigVec=c()

outTab=data.frame()

for(i in rownames(data)){

if(sd(data[i,])<0.001){next}

wilcoxTest=wilcox.test(data[i,] ~ sampleType)

pvalue=wilcoxTest$p.value

if(pvalue<0.05){

Sig=ifelse(pvalue<0.001,"***",ifelse(pvalue<0.01,"**",ifelse(pvalue<0.05,"*","")))

sigVec=c(sigVec, paste0(i, Sig))

conGeneMeans=mean(data[i,1:conNum])

treatGeneMeans=mean(data[i,(conNum+1):ncol(data)])

logFC=log2(treatGeneMeans)-log2(conGeneMeans)

outTab=rbind(outTab,cbind(gene=i,conMean=conGeneMeans,treatMean=treatGeneMeans,logFC=logFC,pValue=pvalue))

}

}

#输出差异分析结果

write.table(outTab, file="diff.xls", sep="\t", row.names=F, quote=F)

write.table(outTab, file="diff.txt", sep="\t", row.names=F, quote=F)

#输出差异基因的表达文件

exp=data[as.vector(outTab[,1]),]

expOut=rbind(ID=colnames(exp), exp)

write.table(expOut, file="diffGeneExp.txt", sep="\t", col.names=F, quote=F)

#绘制差异基因热图

exp=log2(exp+0.1)

row.names(exp)=sigVec

Type=c(rep("Normal",conNum),rep("Tumor",treatNum))

names(Type)=colnames(data)

Type=as.data.frame(Type)

pdf(file="heatmap.pdf", width=9, height=6)

pheatmap(exp,

annotation=Type,

color = colorRampPalette(c(rep("blue",5), "white", rep("red",5)))(50),

cluster_cols =F,

cluster_rows =T,

scale="row",

show_colnames = F,

show_rownames = T,

fontsize = 8,

fontsize_row=8,

fontsize_col=8)

dev.off()

###########TCGA表达数据与生存时间合并

library(limma)

expFile="diffGeneExp.txt"

cliFile="time.txt"

setwd("E:\\ 2")

rt=read.table(expFile,sep="\t",header=T,check.names=F)

rt=as.matrix(rt)

rownames(rt)=rt[,1]

exp=rt[,2:ncol(rt)]

dimnames=list(rownames(exp),colnames(exp))

data=matrix(as.numeric(as.matrix(exp)),nrow=nrow(exp),dimnames=dimnames)

data=avereps(data)

data=data[rowMeans(data)>0,]

group=sapply(strsplit(colnames(data),"\\-"),"[",4)

group=sapply(strsplit(group,""),"[",1)

group=gsub("2","1",group)

data=data[,group==0]

colnames(data)=gsub("(.*?)\\-(.*?)\\-(.*?)\\-(.*?)\\-.*","\\1\\-\\2\\-\\3",colnames(data))

data=t(data)

data=avereps(data)

cli=read.table(cliFile,sep="\t",check.names=F,header=T,row.names=1) ##读取生存时间数据

sameSample=intersect(row.names(data),row.names(cli))

data=data[sameSample,]

cli=cli[sameSample,]

out=cbind(cli,data)

out=cbind(id=row.names(out),out)

write.table(out,file="tcga.expTime.txt",sep="\t",row.names=F,quote=F)

#############单因素cox分析找预后基因及绘制森林图

#引用包

library(survival)

library(survminer)

coxPfilter=0.05 #显著性的过滤标准

inputFile="tcga.expTime.txt" #输入文件

setwd("E:\\ 2") #设置工作目录

#读取输入文件

rt=read.table(inputFile, header=T, sep="\t", check.names=F, row.names=1)

rt$futime=rt$futime/365

#对基因进行循环，查找预后相关的基因

outTab=data.frame()

sigGenes=c("futime","fustat")

for(i in colnames(rt[,3:ncol(rt)])){

#cox分析

cox <- coxph(Surv(futime, fustat) ~ rt[,i], data = rt)

coxSummary = summary(cox)

coxP=coxSummary$coefficients[,"Pr(>|z|)"]

#保留预后相关的基因

if(coxP<coxPfilter){

sigGenes=c(sigGenes,i)

outTab=rbind(outTab,

cbind(id=i,

HR=coxSummary$conf.int[,"exp(coef)"],

HR.95L=coxSummary$conf.int[,"lower .95"],

HR.95H=coxSummary$conf.int[,"upper .95"],

pvalue=coxSummary$coefficients[,"Pr(>|z|)"])

)

}

}

#输出单因素的结果

write.table(outTab,file="TCGA.uniCox.txt",sep="\t",row.names=F,quote=F)

#输出单因素显著基因的表达量

uniSigExp=rt[,sigGenes]

uniSigExp=cbind(id=row.names(uniSigExp),uniSigExp)

write.table(uniSigExp,file="TCGA.uniSigExp.txt",sep="\t",row.names=F,quote=F)

############定义森林图函数############

bioForest=function(coxFile=null,forestFile=null,forestCol=null){

#读取输入文件

rt <- read.table(coxFile,header=T,sep="\t",row.names=1,check.names=F)

gene <- rownames(rt)

hr <- sprintf("%.3f",rt$"HR")

hrLow <- sprintf("%.3f",rt$"HR.95L")

hrHigh <- sprintf("%.3f",rt$"HR.95H")

Hazard.ratio <- paste0(hr,"(",hrLow,"-",hrHigh,")")

pVal <- ifelse(rt$pvalue<0.001, "<0.001", sprintf("%.3f", rt$pvalue))

#输出图形

height=nrow(rt)/12.5+5

pdf(file=forestFile, width = 7,height = height)

n <- nrow(rt)

nRow <- n+1

ylim <- c(1,nRow)

layout(matrix(c(1,2),nc=2),width=c(3,2.5))

#绘制森林图左边的基因信息

xlim = c(0,3)

par(mar=c(4,2.5,2,1))

plot(1,xlim=xlim,ylim=ylim,type="n",axes=F,xlab="",ylab="")

text.cex=0.8

text(0,n:1,gene,adj=0,cex=text.cex)

text(1.5-0.5*0.2,n:1,pVal,adj=1,cex=text.cex);text(1.5-0.5*0.2,n+1,'pvalue',cex=text.cex,font=2,adj=1)

text(3,n:1,Hazard.ratio,adj=1,cex=text.cex);text(3,n+1,'Hazard ratio',cex=text.cex,font=2,adj=1,)

#绘制森林图

par(mar=c(4,1,2,1),mgp=c(2,0.5,0))

xlim = c(0,max(as.numeric(hrLow),as.numeric(hrHigh)))

plot(1,xlim=xlim,ylim=ylim,type="n",axes=F,ylab="",xaxs="i",xlab="Hazard ratio")

arrows(as.numeric(hrLow),n:1,as.numeric(hrHigh),n:1,angle=90,code=3,length=0.05,col="darkblue",lwd=2.5)

abline(v=1,col="black",lty=2,lwd=2)

boxcolor = ifelse(as.numeric(hr) > 1, forestCol[1], forestCol[2])

points(as.numeric(hr), n:1, pch = 15, col = boxcolor, cex=1.6)

axis(1)

dev.off()

}

#调用函数，绘制森林图

bioForest(coxFile="TCGA.uniCox.txt",forestFile="forest.pdf",forestCol=c("red","green"))

####################绘制基因相关性图

#引用包

library(igraph)

library(reshape2)

expFile="diffGeneExp.txt" #表达输入文件

cutoff=0.2 #相关性阈值

out="correlation.pdf" #输出结果文件

setwd("E:\\correlation")

#读取输入文件

data=read.table(expFile, header=T, sep="\t", check.names=F, row.names=1)

#删掉正常样品

group=sapply(strsplit(colnames(data),"\\-"),"[",4)

group=sapply(strsplit(group,""),"[",1)

group=gsub("2","1",group)

rt=data[,group==0]

#计算基因间的相关系数

data=t(rt)

cordata=cor(data)

#保留相关性矩阵的一半

mydata = cordata

upper = upper.tri(mydata)

mydata[upper] = NA

#把相关性矩阵转换为数据框

df = data.frame(gene=rownames(mydata),mydata)

dfmeltdata = melt(df,id="gene")

dfmeltdata = dfmeltdata[!is.na(dfmeltdata$value),]

dfmeltdata = dfmeltdata[dfmeltdata$gene!=dfmeltdata$variable,]

dfmeltdata = dfmeltdata[abs(dfmeltdata$value)>cutoff,]

#定义网络图的节点和边

corweight = dfmeltdata$value

weight = corweight+abs(min(corweight))+5

d = data.frame(p1=dfmeltdata$gene,p2=dfmeltdata$variable,weight=dfmeltdata$value)

g = graph.data.frame(dfmeltdata,directed = FALSE)

#设置颜色，节点大小，字体大小

E(g)$weight = weight

E(g)$color = ifelse(corweight>0,rgb(254/255,67/255,101/255,abs(corweight)),rgb(0/255,0/255,255/255,abs(corweight)))

V(g)$size = 8

V(g)$shape = "circle"

V(g)$lable.cex = 1.2

V(g)$color = "white"

#可视化

pdf(out, width=7, height=6)

layout(matrix(c(1,1,1,0,2,0),byrow=T,nc=3),height=c(6,1),width=c(3,4,3))

par(mar=c(1.5,2,2,2))

vertex.frame.color = NA

plot(g,layout=layout_nicely,vertex.label.cex=V(g)$lable.cex,edge.width = E(g)$weight,edge.arrow.size=0,vertex.label.color="black",vertex.frame.color=vertex.frame.color,edge.color=E(g)$color,vertex.label.cex=V(g)$lable.cex,vertex.label.font=2,vertex.size=V(g)$size,edge.curved=0.4)

#绘制图例

color_legend = c(rgb(254/255,67/255,101/255,seq(1,0,by=-0.01)),rgb(0/255,0/255,255/255,seq(0,1,by=0.01)))

par(mar=c(2,2,1,2),xpd = T,cex.axis=1.6,las=1)

barplot(rep(1,length(color_legend)),border = NA, space = 0,ylab="",xlab="",xlim=c(1,length(color_legend)),horiz=FALSE,

axes = F, col=color_legend,main="")

axis(3,at=seq(1,length(color_legend),length=5),c(1,0.5,0,-0.5,-1),tick=FALSE)

dev.off()

#############################绘制TCGA差异表达HPR热图

library(pheatmap)

conNum=50

treatNum=374

expFile="diffGeneExp.txt"

geneFile="intersectGenes.txt"

setwd("E:\\ pheatmap")

rt=read.table(expFile,header=T,sep="\t",row.names=1,check.names=F)

geneRT=read.table(geneFile,header=F,sep="\t",check.names=F)

hmExp=rt[as.vector(geneRT[,1]),]

hmExp=log2(hmExp+0.1)

Type=c(rep("N",conNum),rep("T",treatNum))

names(Type)=colnames(hmExp)

Type=as.data.frame(Type)

pdf(file="heatmap.pdf",height=6,width=10)

pheatmap(hmExp,

annotation=Type,

color = colorRampPalette(c("blue", "white", "red"))(50),

cluster_cols =F,

show_colnames = F,

show_rownames = T,

scale="row",

fontsize = 12,

fontsize_row=10,

fontsize_col=10)

dev.off()

##############LASSO回归筛选HPG构建cox回归基因

#引用包

library("glmnet")

library("survival")

set.seed(12345)

trainFile="TCGA.uniSigExp.txt" #TCGA数据库输入文件

testFile="icgc.expTime.txt" #ICGC数据库输入文件

setwd("E:\\lasso") #设置工作目录

#读取train组数据文件

rt=read.table(trainFile, header=T, sep="\t", row.names=1)

rt$futime[rt$futime<=0]=0.003

#构建lasso回归模型

x=as.matrix(rt[,c(3:ncol(rt))])

y=data.matrix(Surv(rt$futime,rt$fustat))

fit=glmnet(x, y, family = "cox", maxit = 1000)

#绘制lasso回归图形

pdf("lasso.lambda.pdf")

plot(fit, xvar="lambda", label=TRUE)

dev.off()

#绘制交叉验证图形

cvfit=cv.glmnet(x, y, family="cox", maxit=1000)

pdf("lasso.cvfit.pdf")

plot(cvfit)

abline(v=log(c(cvfit$lambda.min,cvfit$lambda.1se)), lty="dashed")

dev.off()

#找到交叉验证误差最小的点，并且输出模型公式

coef=coef(fit, s = cvfit$lambda.min)

index=which(coef != 0)

actCoef=coef[index]

lassoGene=row.names(coef)[index]

geneCoef=cbind(Gene=lassoGene,Coef=actCoef)

write.table(geneCoef,file="geneCoef.txt",sep="\t",quote=F,row.names=F)

#输出TCGA数据库的风险文件

trainFinalGeneExp=rt[,lassoGene]

myFun=function(x){crossprod(as.numeric(x),actCoef)}

trainScore=apply(trainFinalGeneExp,1,myFun)

outCol=c("futime","fustat",lassoGene)

Risk=as.vector(ifelse(trainScore>median(trainScore),"high","low"))

outTab=cbind(rt[,outCol],riskScore=as.vector(trainScore),Risk)

write.table(cbind(id=rownames(outTab),outTab),file="risk.TCGA.txt",sep="\t",quote=F,row.names=F)

#输出ICGC数据库的风险文件

rt=read.table(testFile, header=T, sep="\t", row.names=1)

rt$futime=rt$futime/365

testFinalGeneExp=rt[,lassoGene]

testScore=apply(testFinalGeneExp,1,myFun)

outCol=c("futime","fustat",lassoGene)

Risk=as.vector(ifelse(testScore>median(testScore),"high","low"))

outTab=cbind(rt[,outCol],riskScore=as.vector(testScore),Risk)

write.table(cbind(id=rownames(outTab),outTab),file="risk.ICGC.txt",sep="\t",quote=F,row.names=F)

###################绘制实验组和验证组生存曲线

#install.packages("survival")

#install.packages("survminer")

#引用包

library(survival)

library(survminer)

setwd("E:\\ survival") #设置工作目录

#定义生存分析的函数

bioSurvival=function(inputFile=null, outFile=null){

#读取输入文件

rt=read.table(inputFile, header=T, sep="\t", check.names=F)

#比较高低风险组的生存差异，得到差异显著性pvalue

diff=survdiff(Surv(futime, fustat) ~ Risk,data = rt)

pValue=1-pchisq(diff$chisq,df=1)

if(pValue<0.001){

pValue="p<0.001"

}else{

pValue=paste0("p=",sprintf("%.03f",pValue))

}

fit <- survfit(Surv(futime, fustat) ~ Risk, data = rt)

#绘制生存曲线

surPlot=ggsurvplot(fit,

data=rt,

conf.int=T,

pval=pValue,

pval.size=6,

legend.title="Risk",

legend.labs=c("High risk", "Low risk"),

xlab="Time(years)",

ylab="Overall survival",

break.time.by = 1,

palette=c("red", "blue"),

risk.table=TRUE,

risk.table.title="",

risk.table.height=.25)

#输出图形

pdf(file=outFile, width=6.5, height=5.5, onefile=FALSE)

print(surPlot)

dev.off()

}

#调用函数,绘制生存曲线

bioSurvival(inputFile="risk.TCGA.txt", outFile="survival.TCGA.pdf")

bioSurvival(inputFile="risk.GEO.txt", outFile="survival.GEO.pdf")

#######################对TCGA和ICGC临床数据多因素COX回归分析

library(survival) #引用包

setwd("E:\\ indep") #设置工作目录

############定义森林图函数############

bioForest=function(coxFile=null, forestFile=null, forestCol=null){

#读取输入文件

rt <- read.table(coxFile, header=T, sep="\t", check.names=F, row.names=1)

gene <- rownames(rt)

hr <- sprintf("%.3f",rt$"HR")

hrLow <- sprintf("%.3f",rt$"HR.95L")

hrHigh <- sprintf("%.3f",rt$"HR.95H")

Hazard.ratio <- paste0(hr,"(",hrLow,"-",hrHigh,")")

pVal <- ifelse(rt$pvalue<0.001, "<0.001", sprintf("%.3f", rt$pvalue))

#输出图形

pdf(file=forestFile, width=6.5, height=4.5)

n <- nrow(rt)

nRow <- n+1

ylim <- c(1,nRow)

layout(matrix(c(1,2),nc=2),width=c(3,2.5))

#绘制森林图左边的临床信息

xlim = c(0,3)

par(mar=c(4,2.5,2,1))

plot(1,xlim=xlim,ylim=ylim,type="n",axes=F,xlab="",ylab="")

text.cex=0.8

text(0,n:1,gene,adj=0,cex=text.cex)

text(1.5-0.5*0.2,n:1,pVal,adj=1,cex=text.cex);text(1.5-0.5*0.2,n+1,'pvalue',cex=text.cex,font=2,adj=1)

text(3.1,n:1,Hazard.ratio,adj=1,cex=text.cex);text(3.1,n+1,'Hazard ratio',cex=text.cex,font=2,adj=1)

#绘制右边的森林图

par(mar=c(4,1,2,1),mgp=c(2,0.5,0))

xlim = c(0,max(as.numeric(hrLow),as.numeric(hrHigh)))

plot(1,xlim=xlim,ylim=ylim,type="n",axes=F,ylab="",xaxs="i",xlab="Hazard ratio")

arrows(as.numeric(hrLow),n:1,as.numeric(hrHigh),n:1,angle=90,code=3,length=0.05,col="darkblue",lwd=3)

abline(v=1, col="black", lty=2, lwd=2)

boxcolor = ifelse(as.numeric(hr) > 1, forestCol, forestCol)

points(as.numeric(hr), n:1, pch = 15, col = boxcolor, cex=2)

axis(1)

dev.off()

}

############定义森林图函数############

#定义独立预后分析函数

indep=function(riskFile=null,cliFile=null,uniOutFile=null,multiOutFile=null,uniForest=null,multiForest=null){

risk=read.table(riskFile, header=T, sep="\t", check.names=F, row.names=1) #读取风险文件

cli=read.table(cliFile, header=T, sep="\t", check.names=F, row.names=1) #读取临床文件

#数据合并

sameSample=intersect(row.names(cli),row.names(risk))

risk=risk[sameSample,]

cli=cli[sameSample,]

rt=cbind(futime=risk[,1], fustat=risk[,2], cli, riskScore=risk[,(ncol(risk)-1)])

#单因素独立预后分析

uniTab=data.frame()

for(i in colnames(rt[,3:ncol(rt)])){

cox <- coxph(Surv(futime, fustat) ~ rt[,i], data = rt)

coxSummary = summary(cox)

uniTab=rbind(uniTab,

cbind(id=i,

HR=coxSummary$conf.int[,"exp(coef)"],

HR.95L=coxSummary$conf.int[,"lower .95"],

HR.95H=coxSummary$conf.int[,"upper .95"],

pvalue=coxSummary$coefficients[,"Pr(>|z|)"])

)

}

write.table(uniTab,file=uniOutFile,sep="\t",row.names=F,quote=F)

bioForest(coxFile=uniOutFile, forestFile=uniForest, forestCol="green")

#多因素独立预后分析

uniTab=uniTab[as.numeric(uniTab[,"pvalue"])<1,]

rt1=rt[,c("futime", "fustat", as.vector(uniTab[,"id"]))]

multiCox=coxph(Surv(futime, fustat) ~ ., data = rt1)

multiCoxSum=summary(multiCox)

multiTab=data.frame()

multiTab=cbind(

HR=multiCoxSum$conf.int[,"exp(coef)"],

HR.95L=multiCoxSum$conf.int[,"lower .95"],

HR.95H=multiCoxSum$conf.int[,"upper .95"],

pvalue=multiCoxSum$coefficients[,"Pr(>|z|)"])

multiTab=cbind(id=row.names(multiTab),multiTab)

write.table(multiTab,file=multiOutFile,sep="\t",row.names=F,quote=F)

bioForest(coxFile=multiOutFile, forestFile=multiForest, forestCol="red")

}

#调用函数，进行独立预后分析

indep(riskFile="risk.TCGA.txt",

cliFile="clinical.txt",

uniOutFile="uniCox.txt",

multiOutFile="multiCox.txt",

uniForest="uniForest.pdf",

multiForest="multiForest.pdf")

###################绘制ROC曲线

#引用包

library(survival)

library(survminer)

library(timeROC)

riskFile="risk.TCGA.txt" #风险文件

cliFile="clinical.txt" #临床数据文件

setwd("E:\\BaiduNetdiskDownload\\铁死亡代码\\铁死亡 - 副本\\22.ROC") #设置工作目录

#读取风险文件

risk=read.table(riskFile, header=T, sep="\t", check.names=F, row.names=1)

risk=risk[,c("futime", "fustat", "riskScore")]

#读取临床数据文件

cli=read.table(cliFile, header=T, sep="\t", check.names=F, row.names=1)

#合并数据

samSample=intersect(row.names(risk), row.names(cli))

risk1=risk[samSample,,drop=F]

cli=cli[samSample,,drop=F]

rt=cbind(risk1, cli)

#定义ROC曲线的颜色

bioCol=rainbow(ncol(rt)-1, s=0.9, v=0.9)

######绘制1 3 5年的ROC曲线######

ROC_rt=timeROC(T=risk$futime, delta=risk$fustat,

marker=risk$riskScore, cause=1,

weighting='aalen',

times=c(1,3,5), ROC=TRUE)

pdf(file="ROC.pdf", width=5.5, height=5.5)

plot(ROC_rt,time=1,col=bioCol[1],title=FALSE,lwd=2)

plot(ROC_rt,time=3,col=bioCol[2],add=TRUE,title=FALSE,lwd=2)

plot(ROC_rt,time=5,col=bioCol[3],add=TRUE,title=FALSE,lwd=2)

legend('bottomright',

c(paste0('AUC at 1 years: ',sprintf("%.03f",ROC_rt$AUC[1])),

paste0('AUC at 3 years: ',sprintf("%.03f",ROC_rt$AUC[2])),

paste0('AUC at 5 years: ',sprintf("%.03f",ROC_rt$AUC[3]))),

col=bioCol[1:3], lwd=2, bty = 'n')

dev.off()

######绘制临床的ROC曲线######

predictTime=5 #定义预测年限

aucText=c()

pdf(file="cliROC.pdf", width=5.5, height=5.5)

#绘制风险得分的ROC曲线

i=3

ROC_rt=timeROC(T=risk$futime,

delta=risk$fustat,

marker=risk$riskScore, cause=1,

weighting='aalen',

times=c(predictTime),ROC=TRUE)

plot(ROC_rt, time=predictTime, col=bioCol[i-2], title=FALSE, lwd=2)

aucText=c(paste0("Risk", ", AUC=", sprintf("%.3f",ROC_rt$AUC[2])))

abline(0,1)

#对临床数据进行循环，绘制临床数据的ROC曲线

for(i in 4:ncol(rt)){

ROC_rt=timeROC(T=rt$futime,

delta=rt$fustat,

marker=rt[,i], cause=1,

weighting='aalen',

times=c(predictTime),ROC=TRUE)

plot(ROC_rt, time=predictTime, col=bioCol[i-2], title=FALSE, lwd=2, add=TRUE)

aucText=c(aucText, paste0(colnames(rt)[i],", AUC=",sprintf("%.3f",ROC_rt$AUC[2])))

}

#绘制图例，得到ROC曲线下的面积

legend("bottomright", aucText,lwd=2,bty="n",col=bioCol[1:(ncol(rt)-1)])

dev.off()

#################绘制风险曲线

library(pheatmap)

setwd("E:\\ riskPlot")

bioRiskPlot=function(inputFile=null,riskScoreFile=null,survStatFile=null){

rt=read.table(inputFile,sep="\t",header=T,row.names=1,check.names=F) #读取输入文件

rt=rt[order(rt$riskScore),]

riskClass=rt[,"risk"]

lowLength=length(riskClass[riskClass=="low"])

highLength=length(riskClass[riskClass=="high"])

lowMax=max(rt$riskScore[riskClass=="low"])

line=rt[,"riskScore"]

line[line>10]=10

pdf(file=riskScoreFile,width = 8,height = 6)

plot(line, type="p", pch=20,

xlab="Patients (increasing risk socre)", ylab="Risk score",

col=c(rep("blue",lowLength),rep("red",highLength)) )

abline(h=lowMax,v=lowLength,lty=2)

legend("topleft", c("High risk", "low Risk"),bty="n",pch=19,col=c("red","blue"),cex=1.2)

dev.off()

color=as.vector(rt$fustat)

color[color==1]="red"

color[color==0]="blue"

pdf(file=survStatFile,width = 8,height = 6)

plot(rt$futime, pch=19,

xlab="Patients (increasing risk socre)", ylab="Survival time (years)",

col=color)

legend("topleft", c("Dead", "Alive"),bty="n",pch=19,col=c("red","blue"),cex=1.2)

abline(v=lowLength,lty=2)

dev.off()

}

bioRiskPlot(inputFile="risk.GEO.txt",riskScoreFile="icgc.riskScore.pdf",survStatFile="icgc.survStat.pdf")

bioRiskPlot(inputFile="risk.TCGA.txt",riskScoreFile="tcga.riskScore.pdf",survStatFile="tcga.survStat.pdf")

###################################绘制nomo图以及校准曲线

#引用包

library(survival)

library(regplot)

library(rms)

riskFile="risk.TCGA.txt" #风险文件

cliFile="tcgaClinical.txt" #临床数据文件

setwd("E:\\ Nomogram ") #设置工作目录

#读取风险输入文件

risk=read.table(riskFile, header=T, sep="\t", check.names=F, row.names=1)

#读取临床数据文件

cli=read.table(cliFile, header=T, sep="\t", check.names=F, row.names=1)

cli=cli[apply(cli,1,function(x)any(is.na(match('unknow',x)))),,drop=F]

cli$Age=as.numeric(cli$Age)

#合并数据

samSample=intersect(row.names(risk), row.names(cli))

risk1=risk[samSample,,drop=F]

cli=cli[samSample,,drop=F]

rt=cbind(risk1[,c("futime", "fustat", "Risk")], cli)

#绘制列线图

res.cox=coxph(Surv(futime, fustat) ~ . , data = rt)

nom1=regplot(res.cox,

plots = c("density", "boxes"),

clickable=F,

title="",

points=TRUE,

droplines=TRUE,

observation=rt[9,],

rank="sd",

failtime = c(1,3,5),

prfail = F)

#输出列线图的风险打分文件

nomoRisk=predict(res.cox, data=rt, type="risk")

rt=cbind(risk1, Nomogram=nomoRisk)

outTab=rbind(ID=colnames(rt), rt)

write.table(outTab, file="nomoRisk.txt", sep="\t", col.names=F, quote=F)

#校准曲线

pdf(file="calibration.pdf", width=5, height=5)

#1年校准曲线

f <- cph(Surv(futime, fustat) ~ Nomogram, x=T, y=T, surv=T, data=rt, time.inc=1)

cal <- calibrate(f, cmethod="KM", method="boot", u=1, m=(nrow(rt)/3), B=1000)

plot(cal, xlim=c(0,1), ylim=c(0,1),

xlab="Nomogram-predicted OS (%)", ylab="Observed OS (%)", lwd=1.5, col="green", sub=F)

#3年校准曲线

f <- cph(Surv(futime, fustat) ~ Nomogram, x=T, y=T, surv=T, data=rt, time.inc=3)

cal <- calibrate(f, cmethod="KM", method="boot", u=3, m=(nrow(rt)/3), B=1000)

plot(cal, xlim=c(0,1), ylim=c(0,1), xlab="", ylab="", lwd=1.5, col="blue", sub=F, add=T)

#5年校准曲线

f <- cph(Surv(futime, fustat) ~ Nomogram, x=T, y=T, surv=T, data=rt, time.inc=5)

cal <- calibrate(f, cmethod="KM", method="boot", u=5, m=(nrow(rt)/3), B=1000)

plot(cal, xlim=c(0,1), ylim=c(0,1), xlab="", ylab="", lwd=1.5, col="red", sub=F, add=T)

legend('bottomright', c('1-year', '3-year', '5-year'),

col=c("green","blue","red"), lwd=1.5, bty = 'n')

dev.off()

################################ssGSEA分析

library(GSVA)

library(limma)

library(GSEABase)

inputFile="symbol.txt"

gmtFile="immune.gmt"

setwd("E:\\ TCGA")

rt=read.table(inputFile,sep="\t",header=T,check.names=F)

rt=as.matrix(rt)

rownames(rt)=rt[,1]

exp=rt[,2:ncol(rt)]

dimnames=list(rownames(exp),colnames(exp))

mat=matrix(as.numeric(as.matrix(exp)),nrow=nrow(exp),dimnames=dimnames)

mat=avereps(mat)

mat=mat[rowMeans(mat)>0,]

geneSet=getGmt(gmtFile, geneIdType=SymbolIdentifier())

ssgseaScore=gsva(mat, geneSet, method='ssgsea', kcdf='Gaussian', abs.ranking=TRUE)

normalize=function(x){

return((x-min(x))/(max(x)-min(x)))}

ssgseaOut=normalize(ssgseaScore)

ssgseaOut=rbind(id=colnames(ssgseaOut),ssgseaOut)

write.table(ssgseaOut,file="ssgseaOut.txt",sep="\t",quote=F,col.names=F)

#############################ssGSEA差异分析

options(stringsAsFactors=F)

library(limma)

library(ggpubr)

library(reshape2)

riskFile="risk.TCGA.txt"

scoreFile="ssgseaOut.txt"

setwd("E:\\ TCGA")

data=read.table(scoreFile,sep="\t",header=T,check.names=F,row.names=1)

group=sapply(strsplit(colnames(data),"\\-"),"[",4)

group=sapply(strsplit(group,""),"[",1)

group=gsub("2","1",group)

data=data[,group==0]

colnames(data)=gsub("(.*?)\\-(.*?)\\-(.*?)\\-(.*?)\\-.*","\\1\\-\\2\\-\\3",colnames(data))

data=avereps(t(data))

risk=read.table(riskFile,header=T,sep="\t",row.names=1,check.names=F)

sameSample=intersect(row.names(data),row.names(risk))

data=data[sameSample,]

risk=risk[sameSample,]

rt=cbind(data,risk[,c("riskScore","risk")])

rt=rt[,-(ncol(rt)-1)]

immCell=c("aDCs","B_cells","CD8+_T_cells","DCs","iDCs","Macrophages",

"Mast_cells","Neutrophils","NK_cells","pDCs","T_helper_cells",

"Tfh","Th1_cells","Th2_cells","TIL","Treg")

rt1=rt[,c(immCell,"risk")]

data=melt(rt1,id.vars=c("risk"))

colnames(data)=c("Risk","Type","Score")

data$Risk=factor(data$Risk, levels=c("low","high"))

p=ggboxplot(data, x="Type", y="Score", color = "Risk",

ylab="Score",add = "none",xlab="",palette = c("blue","red") )

p=p+rotate_x_text(50)

pdf(file="immCell.boxplot.pdf",width=7,height=6)

p+stat_compare_means(aes(group=Risk),symnum.args=list(cutpoints = c(0, 0.001, 0.01, 0.05, 1), symbols = c("***", "**", "*", "ns")),label = "p.signif")

dev.off()

immFunction=c("APC_co_inhibition","APC_co_stimulation","CCR",

"Check-point","Cytolytic_activity","HLA","Inflammation-promoting",

"MHC_class_I","Parainflammation","T_cell_co-inhibition",

"T_cell_co-stimulation","Type_I_IFN_Reponse","Type_II_IFN_Reponse")

rt1=rt[,c(immFunction,"risk")]

data=melt(rt1,id.vars=c("risk"))

colnames(data)=c("Risk","Type","Score")

data$Risk=factor(data$Risk, levels=c("low","high"))

p=ggboxplot(data, x="Type", y="Score", color = "Risk",

ylab="Score",add = "none",xlab="",palette = c("blue","red") )

p=p+rotate_x_text(50)

pdf(file="immFunction.boxplot.pdf",width=7,height=6)

p+stat_compare_means(aes(group=Risk),symnum.args=list(cutpoints = c(0, 0.001, 0.01, 0.05, 1), symbols = c("***", "**", "*", "ns")),label = "p.signif")

dev.off()

####################################绘制风险组在不同临床性状中的生存差异

#引用包

library(survival)

library(survminer)

riskFile="risk.TCGA.txt" #风险输入文件

cliFile="clinical.txt" #临床输入文件

setwd("E:\\ cliGroupSur") #设置工作目录

risk=read.table(riskFile, header=T, sep="\t", check.names=F, row.names=1) #读取风险文件

cli=read.table(cliFile, header=T, sep="\t", check.names=F, row.names=1) #读取临床文件

#数据合并

sameSample=intersect(row.names(cli), row.names(risk))

risk=risk[sameSample,]

cli=cli[sameSample,]

data=cbind(futime=risk[,1],fustat=risk[,2],cli,risk=risk[,"risk"])

#对临床信息进行循环

for(i in colnames(data[,3:(ncol(data)-1)])){

rt=data[,c("futime","fustat",i,"risk")]

rt=rt[(rt[,i]!="unknow"),]

colnames(rt)=c("futime","fustat","clinical","risk")

tab=table(rt[,"clinical"])

tab=tab[tab!=0]

#对每个临床信息里面的分类进行循环

for(j in names(tab)){

rt1=rt[(rt[,"clinical"]==j),]

tab1=table(rt1[,"risk"])

tab1=tab1[tab1!=0]

labels=names(tab1)

if(length(labels)==2){

titleName=j

if((i=="age") | (i=="Age") | (i=="AGE")){

titleName=paste0("age",j)

}

diff=survdiff(Surv(futime, fustat) ~risk,data = rt1)

pValue=1-pchisq(diff$chisq,df=1)

if(pValue<0.001){

pValue="p<0.001"

}else{

pValue=paste0("p=",sprintf("%.03f",pValue))

}

fit <- survfit(Surv(futime, fustat) ~ risk, data = rt1)

#绘制生存曲线

surPlot=ggsurvplot(fit,

data=rt1,

conf.int=F,

pval=pValue,

pval.size=6,

title=paste0("Patients with ",titleName),

legend.title="Risk",

legend.labs=labels,

font.legend=12,

xlab="Time(years)",

break.time.by = 1,

palette=c("red", "blue"),

risk.table=TRUE,

risk.table.title="",

risk.table.col = "strata",

risk.table.height=.25)

#输出图片

j=gsub(">=","ge",j);j=gsub("<=","le",j);j=gsub(">","gt",j);j=gsub("<","lt",j)

pdf(file=paste0("survival.",i,"_",j,".pdf"),onefile = FALSE,

width = 6, #图片的宽度

height =5) #图片的高度

print(surPlot)

dev.off()

}

}

}

########################绘制不同风险组与临床形状差异

#引用包

library(survival)

library(survminer)

riskFile="risk.TCGA.txt" #风险输入文件

cliFile="clinical.txt" #临床输入文件

setwd("E:\\ cliGroupSur") #设置工作目录

risk=read.table(riskFile, header=T, sep="\t", check.names=F, row.names=1) #读取风险文件

cli=read.table(cliFile, header=T, sep="\t", check.names=F, row.names=1) #读取临床文件

#数据合并

sameSample=intersect(row.names(cli), row.names(risk))

risk=risk[sameSample,]

cli=cli[sameSample,]

data=cbind(futime=risk[,1],fustat=risk[,2],cli,risk=risk[,"risk"])

#对临床信息进行循环

for(i in colnames(data[,3:(ncol(data)-1)])){

rt=data[,c("futime","fustat",i,"risk")]

rt=rt[(rt[,i]!="unknow"),]

colnames(rt)=c("futime","fustat","clinical","risk")

tab=table(rt[,"clinical"])

tab=tab[tab!=0]

#对每个临床信息里面的分类进行循环

for(j in names(tab)){

rt1=rt[(rt[,"clinical"]==j),]

tab1=table(rt1[,"risk"])

tab1=tab1[tab1!=0]

labels=names(tab1)

if(length(labels)==2){

titleName=j

if((i=="age") | (i=="Age") | (i=="AGE")){

titleName=paste0("age",j)

}

diff=survdiff(Surv(futime, fustat) ~risk,data = rt1)

pValue=1-pchisq(diff$chisq,df=1)

if(pValue<0.001){

pValue="p<0.001"

}else{

pValue=paste0("p=",sprintf("%.03f",pValue))

}

fit <- survfit(Surv(futime, fustat) ~ risk, data = rt1)

#绘制生存曲线

surPlot=ggsurvplot(fit,

data=rt1,

conf.int=F,

pval=pValue,

pval.size=6,

title=paste0("Patients with ",titleName),

legend.title="Risk",

legend.labs=labels,

font.legend=12,

xlab="Time(years)",

break.time.by = 1,

palette=c("red", "blue"),

risk.table=TRUE,

risk.table.title="",

risk.table.col = "strata",

risk.table.height=.25)

#输出图片

j=gsub(">=","ge",j);j=gsub("<=","le",j);j=gsub(">","gt",j);j=gsub("<","lt",j)

pdf(file=paste0("survival.",i,"_",j,".pdf"),onefile = FALSE,

width = 6, #图片的宽度

height =5) #图片的高度

print(surPlot)

dev.off()

}

}

}

#######################单细胞数据处理

library(stringr)

library(future)

library(future.apply)

library(ggplot2)

library(Seurat)

library(dplyr)

library(ggpubr)

library(future)

library(future.apply)

library(DoubletFinder)

library(harmony)

plan("multicore", workers = 3) ###compute cores

options(future.globals.maxSize = 10000 * 1024^2)

setwd("C:\\ scrna")

inputFile="input.txt"

rt=read.table(inputFile, header=T, sep="\t", check.names=F)

pbmc=CreateSeuratObject(counts =rt,project = "seurat", min.cells=3, min.features=50)

timePoints <- sapply(colnames(pbmc), function(x) unlist(strsplit(x, "\\_"))[1])

timePoints1 <- timePoints

timePoints1<-ifelse(timePoints1 == 'HCC01T', 'T1',

ifelse(timePoints1 == 'HCC02T', 'T2',

ifelse(timePoints1 == 'HCC03N', 'N1',

ifelse(timePoints1 == 'HCC03T', 'T3',

ifelse(timePoints1 == 'HCC04N', 'N2',

ifelse(timePoints1 == 'HCC04T', 'T4',

ifelse(timePoints1 == 'HCC05N', 'N3',

ifelse(timePoints1== 'HCC05T', 'T5',

ifelse(timePoints1 == 'HCC06N', 'N4',

ifelse(timePoints1 == 'HCC06T', 'T6',

ifelse(timePoints1 == 'HCC07N', 'N5',

ifelse(timePoints1 == 'HCC07P', 'P1',

ifelse(timePoints1 == 'HCC07T', 'T7',

ifelse(timePoints1 == 'HCC08N', 'N7',

ifelse(timePoints1 == 'HCC08P', 'P2',

ifelse(timePoints1 == 'HCC08T', 'T8',

ifelse(timePoints1 == 'HCC09N', 'N8',

ifelse(timePoints1 == 'HCC09T', 'T9',

ifelse(timePoints1 == 'HCC10L', 'L1',

ifelse(timePoints1 == 'HCC10N', 'N9', 'T10'))))))))))))))))))))

pbmc <- AddMetaData(object = pbmc, metadata = timePoints1, col.name = 'TimePoints1')

timePoints2 <- timePoints

timePoints2<-ifelse(timePoints2 %in% c('HCC01T', 'HCC02T','HCC03T','HCC04T','HCC05T','HCC06T', 'HCC07T','HCC08T','HCC09T','HCC10T'),'Primary Tumor',

ifelse(timePoints2 %in% c( 'HCC03N', 'HCC04N','HCC05N','HCC06N','HCC07N','HCC08N','HCC09N','HCC10N'),'Nomal Liver',

ifelse(timePoints2 %in% c('HCC07P', 'HCC08P'),'PVTT',' Lymph Node')))

pbmc <- AddMetaData(object = pbmc, metadata = timePoints2, col.name = 'TimePoints2')

pbmc2 <-subset(x =pbmc,TimePoints2 %in% c("Primary Tumor", 'Nomal Liver'))

saveRDS(pbmc2,file = "pbmc2.rds")

data <- readRDS("filename.rds")

#save.image(file="pbmc2.RData")

#load("pbmc2.RData")

#QC 质量控制

#计算血红蛋白hemoglobin基因比例

HB.ref <- c("HBA1","HBA2","HBB","HBD","HBE1","HBG1","HBG2","HBM","HBQ1","HBZ")

rowSums(pbmc[HB.ref,])

HB.genes <- intersect(HB.ref, rownames(pbmc))

HB.genes

#计算每一个特征集的百分比，血红蛋白，线粒体，核糖体

pbmc[["percent.HB"]] <- PercentageFeatureSet(pbmc, features = HB.genes)

pbmc[["percent.MT"]] <- PercentageFeatureSet(pbmc, pattern = "^MT-")

pbmc[["percent.Ribosome"]] <- PercentageFeatureSet(pbmc, pattern = "^RP[SL]")

#画图展示每一个特征集在每个细胞中的百分比

pdf('1_feature_UMI_MT_HB_Ribosome.pdf', width = 30, height = 15)

VlnPlot(pbmc, features = c("nFeature_RNA", "nCount_RNA", "percent.HB", "percent.MT", "percent.Ribosome"), ncol = 5)

dev.off()

#画图展示特征之间的关联性

p1 <- FeatureScatter(pbmc, feature1 = "nCount_RNA", feature2 = "nFeature_RNA")

p2 <- FeatureScatter(pbmc, feature1 = "nCount_RNA", feature2 = "percent.HB")

p3 <- FeatureScatter(pbmc, feature1 = "nCount_RNA", feature2 = "percent.MT")

p4 <- FeatureScatter(pbmc, feature1 = "nCount_RNA", feature2 =

"percent.Ribosome")

pdf('1_feature_relationships.pdf', width = 30, height = 15)

p1 + p2 + p3 + p4

dev.off()

#过滤细胞

#保留检测基因数在200至2500之间的细胞

#保留线粒体基因表达量小于5%的细胞

pbmc<- subset(pbmc,

subset = nFeature_RNA > 500 &

nFeature_RNA < 7000 &

percent.MT < 20

)

pbmc <- NormalizeData(pbmc, normalization.method = "LogNormalize", scale.factor = 10000)

#挑选表达变异度较大的基因

pbmc<- FindVariableFeatures(pbmc, selection.method = "vst", nfeatures = 2000)

# 鉴定前10个表达变异度最大的基因

top10 <- head(VariableFeatures(pbmc), 10)

#可视化高变异度基因

plot1 <- VariableFeaturePlot(pbmc)

plot2 <- LabelPoints(plot = plot1, points = top10, repel = TRUE)

pdf('3_Variable_Features.pdf', width = 12, height = 6)

plot1 + plot2

dev.off()

all.genes <- rownames(pbmc)

scRNA <- ScaleData(pbmc, features = all.genes)

#去掉不需要的特征引入的变异度

pbmc <- ScaleData(pbmc, features = all.genes,

vars.to.regress = c('percent.MT', 'percent.Ribosome',"orig.ident"))

pbmc<- RunPCA(object = pbmc,

features = VariableFeatures(pbmc),

verbose = F,npcs = 50)

#PCA结果展示-1

pdf(paste0("PCA-VizDimLoadings.pdf"),width = 7,height = 5)

VizDimLoadings(pbmc, dims = 1:2, reduction = "pca")

dev.off()

#PCA结果展示-2

pdf(paste0("PCA-DimPlot.pdf"),width = 5,height = 4)

DimPlot(pbmc, reduction = "pca")

dev.off()

#PCA结果展示-3

pdf(paste0("PCA-DimHeatmap.pdf"),width = 5,height = 4)

DimHeatmap(pbmc, dims = 1:6, cells = 500, balanced = TRUE)

dev.off()

pbmc<- RunHarmony(pbmc,

c("orig.ident"),plot_convergence = T)

ggsave(filename = "Harmony_plot_convergence.pdf", width = 10, height = 8)

pdf(paste0("PCA-DimPlot-harmony.pdf"),width = 15,height = 12)

DimPlot(pbmc, reduction = "harmony")

dev.off()

harmony_embeddings <- Embeddings(pbmc, 'harmony')

pbmc=RunTSNE(pbmc,reduction = "harmony", dims = 1:20)

pbmc<- RunUMAP(pbmc,reduction = "harmony", dims =1:20)

pbmc=FindNeighbors(pbmc, reduction = "harmony",

dims = 1:20 )

pbmc=FindClusters(pbmc,

resolution = 0.1)

#### 8. 确定细胞类群分析PC ####

#耗时较久

experiment.aggregate <- JackStraw(experiment.aggregate, num.replicate = 100,dims = 40)

experiment.aggregate <- ScoreJackStraw(experiment.aggregate, dims = 1:40)

pdf(paste0("./",sam.name,"/PCA-JackStrawPlot_40.pdf"),width = 6,height = 5)

JackStrawPlot(object = experiment.aggregate, dims = 1:40)

dev.off()

#碎石图

pdf(paste0("PCA-ElbowPlot.pdf"),width = 6,height = 5)

ElbowPlot(pbmc,ndims = 40)

dev.off()

#确定用于细胞分群的PC

dim.use <- 1:20

#### 9. 细胞分群TSNE算法 ####

#TSNE算法

pbmc<- FindNeighbors(pbmc, dims = dim.use)

pbmc <- FindClusters(pbmc, resolution = 0.5)

pbmc <- RunTSNE(pbmc, dims =1:20,

do.fast = TRUE)

pdf(paste0("CellCluster-TSNEPlot_res0.5PC2.pdf"),width = 20,height = 15)

DimPlot(object = pbmc, pt.size=0.5,label = T,reduction = "tsne")

dev.off()

UMAP算法（选择性运行）

pbmc <- RunUMAP(pbmc, dims = 1:20,do.fast = TRUE)

pdf(paste0("CellCluster-DimPlot_umapres0.5.pdf"),width = 20,height = 15)

DimPlot(pbmc, reduction = "umap")

dev.off()

write.table(pbmc@meta.data,file = paste0("cells_details_umap.txt"))

#按照数据来源分组展示细胞异同--画在一张图中

pdf(paste0("CellCluster-TSNEPlot1.pdf"),width = 25,height = 15)

DimPlot(object = pbmc,

group.by="orig.ident",

pt.size=0.5,reduction = "tsne")

dev.off()

pdf(paste0("CellCluster-UMAPPlot_SamGroup.pdf"),width = 25,height = 15)

DimPlot(object = pbmc,

group.by="orig.ident",

pt.size=0.5,reduction = "umap")

dev.off()

#按照数据来源分组展示细胞异同--画在多张图中

pdf(paste0("CellCluster-TSNEPlot_SamGroup_slipt.pdf"),width = 40,height = 10)

DimPlot(object = pbmc,

split.by ="TimePoints2",

pt.size=0.5,reduction = "tsne")

dev.off()

pdf(paste0("CellCluster-UMAPPlot_SamGroup_slipt.pdf"),width = 40,height = 10)

DimPlot(object = pbmc,

split.by ="TimePoints2",

pt.size=0.5,reduction = "umap")

dev.off()

table(pbmc@meta.data$orig.ident)

#### 10. 计算marker基因 ####

#这一步计算的时候可以把min.pct以及logfc.threshold调的比较低，然后再基于结果手动筛选

all.markers <- FindAllMarkers(pbmc, only.pos = TRUE,

min.pct = 0.3, logfc.threshold = 0.25)

write.table(all.markers,

file=paste0("total_marker_genes.txt"),

sep="\t",quote = F,row.names = F)

#细胞类群相似树

pbmc<- BuildClusterTree(

pbmc,

dims = 1:20,

reorder = F,

reorder.numeric = F)

pdf(paste0("CellCluster-ClusterTree.pdf"),width = 10,height = 8)

PlotClusterTree(pbmc)

dev.off()

save.image( ) = save(list =ls(all=TRUE), file="pbmc1.RData")

#### 细胞类型自动化注释 ####

# 数据来源：http://biocc.hrbmu.edu.cn/CellMarker/download.jsp

library(dplyr)

library(openxlsx)

#### 1. 加载marker数据库 ####

load("./Human_cell_markers.RData")

cellmarker.file <- "./multi/multi_marker_genes_tsne_20PC.txt"

sampleid <- "multi"

#读入单细胞分析中输出的cell marker 基因文件

marker.gene <- read.table(cellmarker.file,header = T,stringsAsFactors = F,sep = "\t")

marker.gene.sig <- marker.gene %>% filter(as.numeric(p_val_adj) <= 0.05)

cat("Totally marker genes:",length(unique(marker.gene.sig$gene)))

table(marker.gene.sig$cluster)

#### 2. 定位数据库中存在的基因 ####

marker.gene.sig %>% filter(gene %in% cell.markers.tb$geneSymbol) -> marker.gene.sel

marker.gene.sel$cellMarker <- apply(marker.gene.sel,1,function(x)paste(unique(cell.markers.tb[cell.markers.tb$geneSymbol == x["gene"],1]),collapse = ","))

cat("Totally find",length(unique(marker.gene.sel$gene)),"/",length(unique(marker.gene.sig$gene)),"genes in cellMarker db")

#### 3. 将marker基因与细胞类型结果写出到文件 ####

write.table(marker.gene.sel,file =paste0("DEG_marker_cells_tsne.txt"),row.names = T,col.names = T,sep = "\t",quote = F)

###################################04.SingleR R包注释细胞类型###################################

counts<-pbmc@assays$RNA@counts

clusters<-pbmc@meta.data$seurat_clusters

ann=pbmc@meta.data$orig.ident

#ref=get(load("ref_Human_all.RData"))

ref=celldex::HumanPrimaryCellAtlasData()

singler=SingleR(test=counts, ref =ref,

labels=ref$label.main, clusters = clusters)

clusterAnn=as.data.frame(singler)

clusterAnn=cbind(id=row.names(clusterAnn), clusterAnn)

clusterAnn=clusterAnn[,c("id", "labels")]

write.table(clusterAnn,file="singleR.clusterAnn.txt",quote=F,sep="\t", row.names=F)

singler2=SingleR(test=counts, ref =ref,

labels=ref$label.main)

cellAnn=as.data.frame(singler2)

cellAnn=cbind(id=row.names(cellAnn), cellAnn)

cellAnn=cellAnn[,c("id", "labels")]

write.table(cellAnn, file="singleR.cellAnn.txt", quote=F, sep="\t", row.names=F)

#cluster注释后的可视化

newLabels=singler$labels

names(newLabels)=levels(pbmc)

pbmc=RenameIdents(pbmc, newLabels)

pdf(file="04.TSNE-rename.pdf",width=20,height=15)

TSNEPlot(object = pbmc, pt.size = 2, label = TRUE) #TSNE可视化

dev.off()

pdf(file="04.UMAP-rename.pdf",width=20,height=15)

UMAPPlot(object = pbmc, pt.size = 2, label = TRUE) #TSNE可视化

dev.off()

write.table(pbmc@metadata, file="metadata.txt", quote=F, sep="\t", row.names=F)

pbmc$celltype1 <- Idents(pbmc)

Idents(pbmc) <- "TimePoints2"

pbmc2 <- subset(pbmc, idents = "Primary Tumor")

logFCfilter=1 #logFC的过滤条件

adjPvalFilter=0.05

sig.markers=all.markers[(abs(as.numeric(as.vector(all.markers$avg_log2FC)))>logFCfilter & as.numeric(as.vector(all.markers$p_val_adj))<adjPvalFilter),]

write.table(sig.markers,file="03.clusterMarkers.txt",sep="\t",row.names=F,quote=F)

###################################05.monocle R包细胞轨迹分析###################################

#准备细胞轨迹分析需要的文件

monocle.matrix=as.matrix(pbmc2@assays$RNA@data)

monocle.sample=pbmc2@meta.data

monocle.geneAnn=data.frame(gene_short_name = row.names(monocle.matrix), row.names = row.names(monocle.matrix))

monocle.clusterAnn=clusterAnn

monocle.markers=sig.markers

#将Seurat结果转换为monocle需要的细胞矩阵，细胞注释表格和基因注释表格

data <- as(as.matrix(monocle.matrix), 'sparseMatrix')

pd<-new("AnnotatedDataFrame", data = monocle.sample)

fd<-new("AnnotatedDataFrame", data = monocle.geneAnn)

cds <- newCellDataSet(data, phenoData = pd, featureData = fd)

names(pData(cds))[names(pData(cds))=="seurat_clusters"]="Cluster"

pData(cds)[,"Cluster"]=paste0("cluster",pData(cds)[,"Cluster"])

#添加细胞聚类数据

clusterAnn=as.character(monocle.clusterAnn[,2])

names(clusterAnn)=paste0("cluster",monocle.clusterAnn[,1])

pData(cds)$cell_type2 <- plyr::revalue(as.character(pData(cds)$Cluster),clusterAnn)

#细胞轨迹分析流程

cds <- estimateSizeFactors(cds)

cds <- estimateDispersions(cds)

cds <- setOrderingFilter(cds, as.vector(sig.markers$gene))

#plot_ordering_genes(cds)

cds <- reduceDimension(cds, max_components = 2, reduction_method = 'DDRTree')

cds <- orderCells(cds)

#保存树枝的细胞轨迹图

pdf(file="05.trajectory.State.pdf",width=6.5,height=6)

plot_cell_trajectory(cds,color_by = "State")

dev.off()

#保存时间的细胞轨迹图

pdf(file="05.trajectory.Pseudotime.pdf",width=6.5,height=6)

plot_cell_trajectory(cds,color_by = "Pseudotime")

dev.off()

#保存细胞名称的细胞轨迹图

pdf(file="05.trajectory.cellType.pdf",width=6.5,height=6)

plot_cell_trajectory(cds,color_by = "cell_type2")

dev.off()

#保存聚类的细胞轨迹图

pdf(file="05.trajectory.cluster.pdf",width=6.5,height=6)

plot_cell_trajectory(cds, color_by = "Cluster")

dev.off()

#细胞轨迹差异分析

groups=subset(pData(cds),select='State')

pbmc=AddMetaData(object=pbmc, metadata=groups, col.name="group")

geneList=list()

for(i in levels(factor(groups$State))){

pbmc.markers=FindMarkers(pbmc, ident.1 = i, group.by = 'group')

sig.markers=pbmc.markers[(abs(as.numeric(as.vector(pbmc.markers$avg_log2FC)))>logFCfilter & as.numeric(as.vector(pbmc.markers$p_val_adj))<adjPvalFilter),]

sig.markers=cbind(Gene=row.names(sig.markers), sig.markers)

write.table(sig.markers,file=paste0("05.monocleDiff.", i, ".txt"),sep="\t",row.names=F,quote=F)

geneList[[i]]=row.names(sig.markers)

}

#保存交集基因

unionGenes=Reduce(union,geneList)

write.table(file="05.monocleDiff.union.txt",unionGenes,sep="\t",quote=F,col.names=F,row.names=F)

pbmc<- RenameIdents(

object = pbmc,

"0" = "CD4+ cytotoxic T cell",

"1" = "Kupffer cell",

"2" = "Liver bud hepatic cell",

"3" = "CD8+ T cell",

"4" = "Endothelial cell",

"5" = "Monocyte",

"6" = "Liver bud hepatic cell",

"7" = "CD8+ T cell",

"8" = "Myofibroblast",

"9" = "CD4+ cytotoxic T cell",

"10" = "Hepatocyte",

"11" = "Exhausted CD4+ T cell",

"12" = "B cell",

"13" = "Hepatocyte",

"14" = "B cell",

"15" = "Dendritic cell",

"16" = "Hepatocyte",

"17" = "Exhausted CD8+ T cell",

"18" = "Cancer stem cell",

"19" = "Liver bud hepatic cell",

"20" = "Endothelial cell",

"21" = "Liver bud hepatic cell",

"22" = "Hepatocyte",

"23" = "Kupffer cell",

"24" = "Liver bud hepatic cell"

)

pbmc$celltype.group <- paste(Idents(pbmc), pbmc$TimePoints2, sep = "_")

pdf(paste0("CellCluster-slipt-time-tsne","PC.pdf"),width = 12,height = 15)

DimPlot(pbmc,

split.by ="TimePoints2",

reduction = "tsne",label = F,raster=FALSE)

dev.off()

pdf(paste0("CellCluster-slipt-time-umap","PC.pdf"),width = 12,height = 15)

DimPlot(pbmc,

split.by ="TimePoints2",

reduction = "umap",label = F,raster=FALSE)

dev.off()

pdf(paste0("CellClusterAll-group.pdf"),width = 20,height =8)

DimPlot(object = pbmc,

group.by="TimePoints2",

pt.size=0.5,reduction = "tsne",raster=FALSE)

dev.off()

pdf(paste0("CellClusterAll-group-umap.pdf"),width = 12,height =8)

DimPlot(object = pbmc,

group.by="TimePoints2",

pt.size=0.5,reduction = "umap",raster=FALSE)

dev.off()

pdf(paste0("CellClusterAll-group-tsne.pdf"),width = 12,height =8)

DimPlot(object = pbmc,

group.by="TimePoints2",

pt.size=0.5,reduction = "tsne",raster=FALSE)

dev.off()

pdf(paste0("CellCluster-sliptumap3.pdf"),width = 16,height = 8)

DimPlot(pbmc,

split.by ="TimePoints2",

reduction = "umap",label = F,raster=FALSE)

dev.off()

pdf(paste0("CellCluster-slipttsne3.pdf"),width = 16,height = 8)

DimPlot(pbmc,

split.by ="TimePoints2",

reduction = "tsne",label = F,raster=FALSE)

dev.off()

pdf(paste0("CellClusterAll-group4.pdf"),width = 12,height =10)

DimPlot(object = pbmc,

group.by="TimePoints2",

pt.size=0.5,reduction = "tsne",label=pbmc$celltype,raster=FALSE)

dev.off()

FeaturePlot(pbmc,feature ="INCENP",reduction = "umap",label = F,raster=FALSE)

VlnPlot(pbmc,feature = "INCENP",pt.size = 0)

INCENP

NEK11

AURKB

CCNA2

PRKAA2

CHEK1

CDK5

PRKCD

CDK1

vargene<-pbmc[["RNA"]]@var.features

write.table(file="vargene.txt",vargene,sep="\t",quote=F,col.names=F,row.names=F)

RNAgene<-pbmc@assays$RNA

#将seurat对象转换成monocle对象

##############################

data <- pbmc2[['RNA']]@data

pd <- new('AnnotatedDataFrame', data = pbmc2@meta.data)

fData <- data.frame(gene_short_name = row.names(data), row.names = row.names(data))

fd <- new('AnnotatedDataFrame', data = fData)

HSMM <- newCellDataSet(data,

phenoData = pd,

featureData = fd

)

#########################

#构建细胞发育轨迹

#########################

ordering_genes <- pbmc2[["RNA"]]@var.features

HSMM <- setOrderingFilter(HSMM, ordering_genes)

HSMM <- estimateSizeFactors(HSMM)

#降维

HSMM <- reduceDimension(HSMM,

norm_method="none",

reduction_method="DDRTree",

max_components=3,

scaling=TRUE,

verbose=TRUE,

pseudo_expr=0)

#将细胞摆放到轨迹上

HSMM <- orderCells(HSMM)

#绘图展示

#以state来展示

pdf('trajectory_state.pdf')

plot_cell_trajectory(HSMM)

dev.off()

#以细胞类型来展示

pdf('trajectory_celltype.pdf')

plot_cell_trajectory(HSMM, color_by = 'celltype')

dev.off()

#分别展示每一个细胞类型的发育轨迹

pdf('trajectory_celltype_separate.pdf')

plot_cell_trajectory(HSMM, color_by = 'celltype')+ facet_wrap(~celltype, nrow = 2) + NoLegend()

dev.off()

pdf('trajectory_celltype_separate2.pdf')

plot_cell_trajectory(HSMM, color_by = 'celltype')+ facet_wrap(~celltype, nrow = 2)

dev.off()

#查看特定的gene在发育轨迹上的表达情况

pdf("5gene_trajectory.pdf",width = 20,height =20)

plot_cell_trajectory(HSMM,markers=c("INCENP","NEK11","AURKB","CCNA2","PRKAA2","CHEK1

","CDK5","PRKCD","CDK1"),use_color_gradient=T)

dev.off()

siggene<-c("INCENP","NEK11","AURKB","CCNA2","PRKAA2","CHEK1","CDK5","PRKCD","CDK1")

#heatmap

top5=scRNA.markers %>% group_by(cluster) %>% top_n(n = 5, wt = avg_log2FC)

sig_gene_names <- unique(top5$gene)

pseudotemporalplot<- plot_pseudotime_heatmap(HSMM[siggene,],

num_clusters = 2, #亚群数需要对应修改

cores = 4,

hmcols = NULL,

show_rownames = T,

return_heatmap = T)

pdf(file="monocle_heatmap.pdf")

pseudotemporalplot

dev.off()

pbmc2 <-subset(x =pbmc,celltype %in% c("Hepatocyte", 'Cancer stem cell'))

plot_cell_trajectory(HSMM, markers = c("VCAN","CD163","MRC1"),use_color_gradient = T,show_branch_points = F)

pseudotemporalplot<- plot_pseudotime_heatmap(HSMM[siggene,],

num_clusters = 2, #亚群数需要对应修改

cores = 4,

hmcols = colorRampPalette(rev(brewer.pal(9, "PRGn")))(62),

show_rownames = T,

return_heatmap = T)

pdf(file="monocle_heatmap2.pdf")

pseudotemporalplot

dev.off()
